# STARR-seq identifies active, chromatin-masked, and dormant enhancers in pluripotent mouse embryonic stem cells

**DOI:** 10.1186/s13059-020-02156-3

**Published:** 2020-09-10

**Authors:** Tianran Peng, Yanan Zhai, Yaser Atlasi, Menno ter Huurne, Hendrik Marks, Hendrik G. Stunnenberg, Wout Megchelenbrink

**Affiliations:** 1 Department of Molecular Biology, Radboud Institute for Molecular Life Sciences, Radboud University, Geert Grooteplein Zuid 28, 6525 GA Nijmegen, The Netherlands; 2 Princess Máxima Center for Pediatric Oncology, Heidelberglaan 25, 3584 CS Utrecht, The Netherlands; 3 Department of Precision Medicine, University of Campania Luigi Vanvitelli, Vico L. De Crecchio 7, 80138 Naples, Italy

## Abstract

**Background:**

Enhancers are distal regulators of gene expression that shape cell identity and control cell fate transitions. In mouse embryonic stem cells (mESCs), the pluripotency network is maintained by the function of a complex network of enhancers, that are drastically altered upon differentiation. Genome-wide chromatin accessibility and histone modification assays are commonly used as a proxy for identifying putative enhancers and for describing their activity levels and dynamics.

**Results:**

Here, we applied STARR-seq, a genome-wide plasmid-based assay, as a read-out for the enhancer landscape in “ground-state” (2i+LIF; 2iL) and “metastable” (serum+LIF; SL) mESCs. This analysis reveals that active STARR-seq loci show modest overlap with enhancer locations derived from peak calling of ChIP-seq libraries for common enhancer marks. We unveil ZIC3-bound loci with significant STARR-seq activity in SL-ESCs. Knock-out of Zic3 removes STARR-seq activity only in SL-ESCs and increases their propensity to differentiate towards the endodermal fate. STARR-seq also reveals enhancers that are not accessible, masked by a repressive chromatin signature. We describe a class of dormant, p53 bound enhancers that gain H3K27ac under specific conditions, such as after treatment with Nocodazol, or transiently during reprogramming from fibroblasts to pluripotency.

**Conclusions:**

In conclusion, loci identified as active by STARR-seq often overlap with those identified by chromatin accessibility and active epigenetic marking, yet a significant fraction is epigenetically repressed or display condition-specific enhancer activity.

## Introduction

Mouse embryonic stem cells (mESCs) derived from the inner cell mass of the early developing embryo can propagate indefinitely in vitro and are pluripotent [1–3]. Pluripotent stem cells can give rise to all somatic cell lineages, a fundamental property for the development of complex organisms, including humans, that holds great promise for regenerative medicine [4–6]. Murine ESCs cultured in medium supplemented with serum and leukemia inhibitory factor (serum + LIF; SL) are metastable and prone to spontaneous differentiation [7, 8]. Culturing in serum-free medium supplemented with two small kinase inhibitors and LIF (2i + LIF; 2iL), however, results in more homogenous cell populations that bear greater similarity to the inner cell mass of the preimplantation epiblast and better recapitulate the pluripotent “ground state” [9–12]. SL and 2iL-cultured cells are both naïve, in contrast to stem cells (EpiLCs) that are pluripotent but “primed” for lineage specification [13–15]. Despite the functional similarity between 2iL- and SL ESCs, they are profoundly different in their metabolic, epigenetic, and transcriptional state exemplified by over 1500 differentially expressed genes [10, 16–22]. Importantly, the two naïve pluripotent states can be interconverted by simply switching the culture medium, making 2iL- vs SL-ESCs an attractive model system to study principles of gene regulation [23, 24].

It is well appreciated that gene expression is regulated by a complex network of regulatory elements that promote (promoters) and enhance (enhancers) RNA transcription through proximal and distal binding of key transcription factors (TFs), respectively [25]. Given their key role in gene regulation, enhancers have been studied in many cell types, including murine and human ESCs. However, large-scale direct measurements of tissue-specific enhancer function are time-consuming [26–28]. Alternatively, putative enhancers can be recognized by the presence of specific TFs and accessible chromatin marked by several histone modifications that are associated with active regulatory elements, such as H3K27ac. Profiling studies resulted in the notion of thousands of enhancers that are frequently grouped near key cell identity genes [29]. Other studies used chromatin accessibility, sets of histone marks, or TFs binding to detect enhancers that are specific for the pluripotent ground- (2iL), metastable (SL), or primed (EpiLC) state [18, 30–34].

Despite significant progress, it remains difficult to estimate enhancer potency from epigenetic marking. For example, “poised enhancers,” that are occupied by P300 and establish significant interactions with promoters of lineage specifying genes in ESCs, are repressed by H3K27me3 [35]. Seed enhancers are occupied by low levels of H3K4me1, and in half of the cases by H3K27ac, but become fully active at later developmental stages [30]. Furthermore, focusing on accessible chromatin or common histone modifications to pinpoint enhancers, fails to identify enhancers lacking such common enhancer marks [36]. An alternative way of testing enhancer activity is by employing massively parallel reporter assays that measure enhancer activity by combining genomic fragments and a minimal promoter and transfecting the constructs into cells [37]. The self-transcribing active regulatory region sequencing [38] (STARR-seq) method is particularly attractive, as it allows quantitative measurement of enhancer strength in a genome-wide fashion by high-throughput sequencing. Genome-wide STARR-seq has first been applied to Drosophila [38–40] and later also in mammalian cells [41, 42]. Targeted STARR-seq approaches use enrichment strategies (e.g., genomic fragments precipitated in ChIP-seq) to construct comprehensive libraries of candidate enhancer loci. This technique has been employed to examine enhancer activity in mammalian cells [43], including human embryonic stem cells [44].

Here, we applied whole genome STARR-seq in mESC cultured in 2iL or SL. Comparing genome-wide quantitative STARR-seq active loci and chromatin marking reveals a class of SL-specific enhancers located near naïve and primed pluripotency genes that are activated in naked DNA by ZIC3, but remain repressed within the chromatin context of SL-ESCs. Furthermore, we detected P53-driven enhancers with strong STARR-seq signal, but very low chromatin accessibility and histone marking. These enhancers gain H3K27ac upon treatment with Nocodazole or transiently during reprogramming. Taken together, our STARR-seq assay reveals active, dormant, and chromatin masked enhancers.

## Results

### Defining a robust set of STARR-seq enhancers in 2iL- and SL-ESCs

**Fig. 1:**
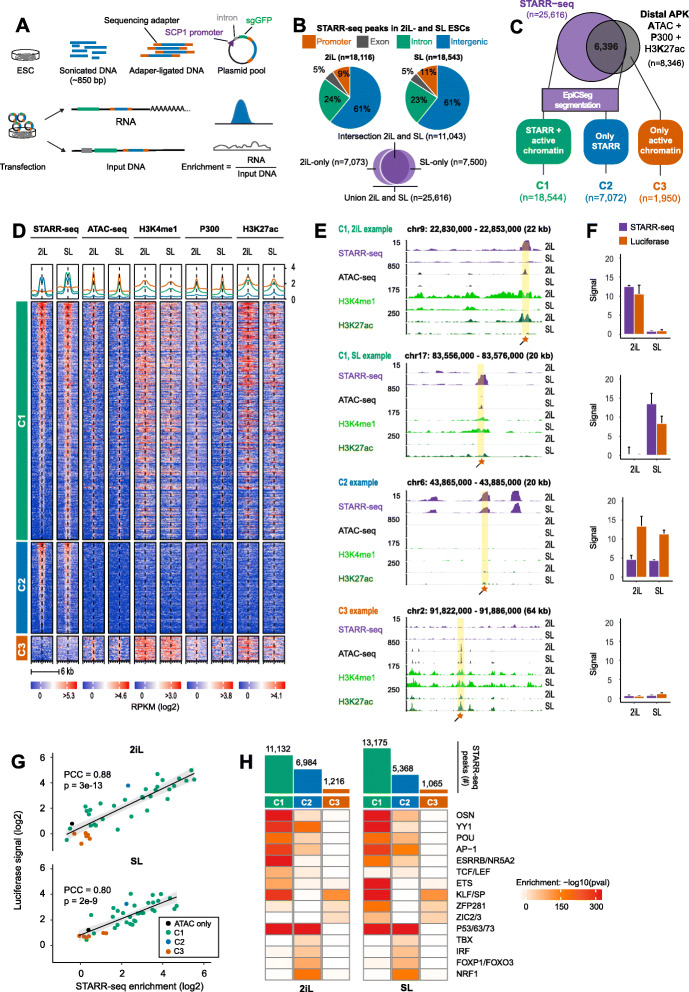
Comparison of genome-wide STARR-seq and active chromatin in mouse ESCs defines three classes of regulatory elements. **a** Experimental setup of genome-wide STARR-seq. ESC DNA is sonicated into random fragments with a median size of 850 bp. Adapter-ligated fragments are cloned behind the SCP minimal promoter and transfected into ESCs. STARR-seq signal represents the number of transcribed fragments divided by sequenced input DNA in a “peak” region. Bayesian shrinkage was applied to penalize the signal of loci with low read counts (see methods). **b** Genomic distribution of the significant STARR-seq peaks (enrichment ≥ 3, *p* < 0.05) detected in 2iL or SL. 18,116 enhancers were detected in 2iL and 18,543 in SL. 7073 enhancers were only detected in 2iL and 7500 enhancers were only detected in SL. The union of STARR-seq peaks detected in 2iL- and SL comprises 25,616 enhancer loci. **c** Top: Putative enhancers were defined as the intersection of ATAC-seq, P300, and H3K27ac ChIP-seq peaks. For each mark, the peaks found in the union of 2iL- and SL-ESCs were taken. The union of all the APK loci (present in 2iL or SL) and all the STARR-seq peaks (2iL or SL) were classified by EpiCSeg (see methods). Bottom: We defined loci as C1: STARR-seq and active chromatin, C2: only STARR-seq or C3: active chromatin, but no STARR-seq. C3-loci near a Gencode or Refseq TSS were discarded. [PD]. **d** Heatmap of STARR-seq and enhancer marks in 2iL- and SL-ESCs. Signal was computed on the STARR- (C1 and C2) or ATAC-seq peak (C3) flanked by 3 kb. Regions were clustered by class and ranked by decreasing STARR-seq signal (log_2_ RPKM) in 2iL. The signal intensity (log_2_ RPKM) was capped at 75% of the maximum value to enhance visualization. [PD]. **e** Genome browser view for selected C1- (top), C2- (middle), and C3- (bottom) loci. The STARR-seq track depicts the enrichment over input. For the other tracks, the signal is shown in RPKM. Orange boxes denote luciferase regions (see Table S3 for primers and locations). [PD]. **f** STARR-seq and luciferase signal for regions shown in **e**. Luciferase signal is defined as the Firefly over Renilla (F/R) scaled to the F/R value of an empty vector. These values were log_2_ transformed and linearly scaled to STARR-seq log_2_ enrichment values (see the “Methods” section). Error bars denote the standard deviation for biological duplicates (STARR-seq) or technical triplicates (luciferase). **g** STARR-seq enrichment and luciferase signal (as in **f**) for *n* = 39 selected loci in 2iL- and SL-ESCs. Points denote the mean of biological duplicates (STARR-seq) or technical triplicates (luciferase). PCC: Pearson’s correlation coefficient. **h** Enrichment of known DNA motifs at C1-, C2-, and C3-loci relative to a GC%-, size-, and input-matched background set (see the “Methods” section). The bars (top) depict the number of STARR-seq peaks detected per class. Both the class definition and the STARR-seq peaks are condition-specific (2iL or SL). TFs with similar motifs were grouped. *P* values: Homer2 binomial test with Benjamini-Hochberg correction. *Some of the panels in these figures contain public data. These panels are annotated with [PD]. The accession numbers of public data and their corresponding panels are annotated in* Additional file 2: Table S1

We applied whole genome STARR-seq to quantify and compare the enhancer sets in 2iL- and SL-ESCs (Fig. 1a). In brief (see the “Methods” section for details), genomic DNA was sonicated into segments of ~ 850 bp to maximize the chance of obtaining complete enhancers. Next, fragments were adapter-ligated and cloned after a minimal Super Core Promoter (SCP1). Plasmids were transfected into 2iL- and SL-cultured mESCs, and the number of transcripts originating from the minimal SCP1 promoter was quantified by high-throughput sequencing. Simultaneously, the number of transfected DNA fragments (input DNA) was quantified by sequencing. Input DNA covered 83% of the mappable genome with a mean coverage of 9.2x (Additional file 1: Fig. S1A). After alignment of STARR-seq and input DNA, initial STARR-seq “peaks” were called using the MACS2 peak caller [45]. Next, a binomial model was used to further assess the significance of all these initially detected STARR-seq peaks (union of 2iL and SL) in each of the four STARR-seq libraries generated. Finally, STARR-seq enrichment, defined as the fraction of STARR-seq reads per input read was computed at each peak and corrected for low counts using Bayesian shrinkage. The same procedure was used for 1 million GC% and size-matched regions, of which less than 3% exceeded a 3-fold enrichment (Additional file 1: Fig. S1B). Therefore, we defined the final STARR-seq peaks as MACS2 peaks exceeding a 3-fold enrichment with binomial *p* value < 0.05 in both biological replicates, which included a total of 25,616 peaks. These STARR-seq peaks were reproducible between biological replicates and mostly located in intronic and intergenic loci (Additional file 1: Fig. S1C; Fig. 1b). Finally, input- and STARR-seq libraries had no specific GC% bias and subsampling of STARR-seq and input reads indicated approximate convergence of the expected number of enhancers at the applied sequencing depth (Additional file 1: Fig. S1D-E).

### Defining three classes of putative regulatory elements

Having determined a robust set of STARR-seq peaks, we assessed to what extent they overlap promoter distal loci with accessible DNA (ATAC-seq), occupied by P300 and flanked by H3K27ac (henceforth APK-elements), a chromatin signature associated with active regulatory elements [46]. This analysis showed that 75% (19,220 of 25,616) of our STARR-seq peaks are not intersecting an APK-element (Fig. 1c, upper diagram). Therefore, we decided to thoroughly examine evidence of chromatin accessibility, TF occupancy, or histone modifications at all our STARR-seq peaks. To this end, we segmented the mouse genome with EpiCSeg [47] using 22 ChIP-seq data sets collected in 2iL- and SL-ESCs (Fig. 1c lower panel; Additional file 1: Fig. S1F; Additional file 2: Table S1). This showed that the majority of the STARR-seq peaks (*n* = 18,544) in fact overlap genomic loci tentatively labeled as “active” enhancers, henceforth referred to as class C1-loci. Still, 7072 STARR-seq peaks do not display an active chromatin signature, but possess enhancer activity in the reporter assay suggesting that these are inactive/masked in a chromatin context (termed C2-loci). Finally, 1950 APK-elements displayed no STARR-seq signal in any of the four replicates (*p* > 0.1; C3-loci; Fig. 1c, d). Enhancer activity at C1-, C2- and C3-loci was validated by luciferase reporter assays (Fig. 1e–g, Additional file 3: Table S2). Chromatin accessibility (Additional file 1: Fig. S1G) and H3K27ac (Additional file 1: Fig. S1H) correlate poorly (*r* < 0.3) with STARR-seq enrichment at C1-loci, indicating that enhancer strength cannot be easily predicted from these chromatin features (Additional file 1: Fig. S1I).

Next, we set out to determine which TFs were associated with C1-, C2-, and C3-loci. To this end, we computed enrichment for TF motifs in each class relative to motifs found in a matched background set (methods; Fig. 1h). As expected, C1 enhancers are specifically enriched for well-known pluripotency factors like OCT4, SOX2, NANOG (together abbreviated as OSN), ESRRB, and KLF4, as well as more general TFs like AP-1 and YY1. C2-loci are highly enriched for P53, NRF1, and Forkhead motifs. Finally, C3-loci are highly enriched for GC-rich zinc finger motifs like KLF/SP and the (primed) pluripotency factors ZIC2/3, but lack motifs of the OSN core pluripotency factors. We detected enrichment for TCF/LEF motifs only at STARR-seq peaks in 2iL-cultured ESCs. ZIC2/3 motifs on the other hand are only enriched at STARR-seq peaks of SL-cultured ESCs (Fig. 1h).

We next assessed the C3-loci that are distal from annotated Gencode and Refseq transcription start sites. These loci displayed no significant STARR-seq signal despite the presence of an active chromatin signature. The lack of enhancer activity is not due to low coverage of input DNA fragments at C3-loci, which is equal or even higher compared to the other two classes (Additional file 1: Fig. S1J). Comparing the GC% per class revealed that C3-loci are significantly more GC-rich compared to C1- or C2-loci (Additional file 1: Fig. S1K; *p* < 2e-16, Wilcoxon rank-sum test) and the fraction of C3-loci that overlap a CpG island is higher (8%) compared to C1- (5%) or C2-loci (< 1%, Additional file 1: Fig. S1L). These results indicate that C3-loci might be enriched for promoter regions of unannotated transcripts. To test this hypothesis, we overlapped C3-loci with transcription start sites defined by the Phantom5 consortium using Cap Analysis of Gene Expression (CAGE) [48, 49] and found that 60% of the C3-loci coincide with a CAGE peak. Thus, many C3-loci overlap with unannotated transcripts that have an active chromatin signature, but do not elicit STARR-seq enhancer activity (Additional file 1: Fig. S1M).

In short, STARR-seq combined with epigenetic analysis revealed three classes of enhancers: C1 STARR-seq peaks that are accessible and marked by a chromatin signature associated typically with active enhancers, C2-loci that reside in largely inaccessible DNA that is void of a classic active chromatin signature and C3-loci that are accessible and do have an active chromatin signature, but have no significant STARR-seq signal and frequently overlap promoters.

### Class C1 STARR-seq loci recapitulate ESC enhancer dynamics

**Fig. 2:**
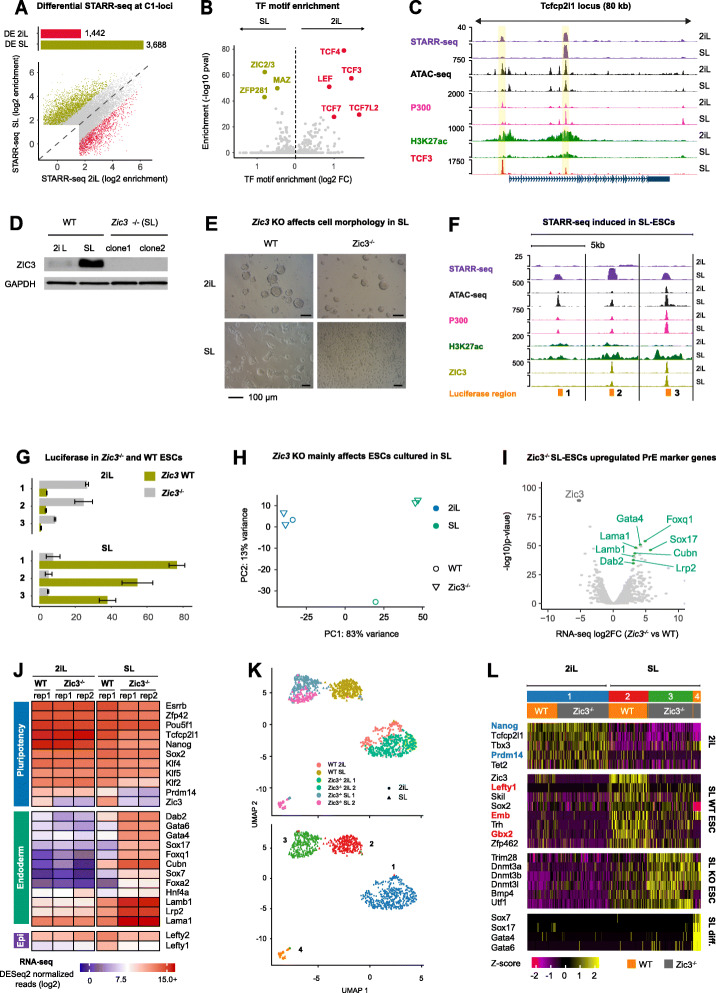
ZIC3 is required for enhancer activity in SL-ESCs, but not in 2iL-ECSs. **a** Top: Barplot that depicts the number of C1 STARR-seq peaks (*n* = 18,544) with significantly higher (FC ≥ 2.5, *p* < 0.05, DESeq2) enrichment in 2iL (red; *n* = 1442) or SL (green; *n* = 3688). Bottom: Scatterplot of the STARR-seq enrichment in 2iL or SL for C1 STARR-seq peaks. Differential peaks elevated in 2iL (red) or SL (green) are annotated. Non-differential peaks are gray. **b** TF motifs enriched at differential C1 STARR-seq peaks in 2iL- (red) and SL-ESCs (green). *P* values were derived using a binomial test with all C1 STARR-seq peaks as background set (Homer2). **c** STARR-seq peaks (highlighted in yellow) near the *Tcfcp2l1* gene that is higher expressed in 2iL-ESCs [50]. A STARR-seq enhancer near the TSS is repressed by TCF3 in SL-ESCs [PD]. **d** Western blot analysis of ZIC3 and a GAPDH control in 2iL- and SL-WT ESCs (left) and two *Zic3*^−/−^ ESC clones cultured in SL (right). **e** Cell morphology of *Zic3*^−/−^ and WT ESCs cultured in 2iL or SL. Zic3^−/−^ ESCs drastically change morphology. **f** Examples of STARR-seq peaks with elevated signal in SL-ESCs. Sites are more accessible in SL-ESCs and have slightly higher H3K27ac, although this signal is relatively low (see, e.g., Figure S1I). P300 ChIP-seq signal is similar between 2iL- and SL-ESCs [PD]. **g** Luciferase signal (Firefly/Renilla) scaled to F/R of a control region in WT and *Zic3*^−/−^ ESCs for the three genomic locations shown in **f**. See Table S3 for genomic location and primer sequences. **h** PCA plot using the 1000 most variable genes in *Zic3* WT vs *Zic3*^−/−^ RNA-seq. Loss of *Zic3* barely affects the transcriptome of 2iL-ESCs, but alters that of SL-ESCs considerably. **i** Differentially expressed genes in *Zic3*^−/−^ vs WT ESCs cultured in SL. Strongly upregulated genes are enriched for endodermal markers like *Sox17*, *Gata4*, and *Dab2* and *Cubn*. **j** RNA-seq expression of selected pluripotency factors and endodermal marker genes. The color scale denotes DEseq2 normalized reads (log_2_) per gene. Biological duplicates are shown for *Zic3*^−/−^ ESCs. **k** UMAP clustering of *Zic3*^−/−^ and WT ESC cultured in 2iL and SL. Top: annotation of genotype and culture condition. Bottom: cluster assignment using shared nearest neighbors on the first 12 principal components and resolution = 0.1. **l** Heatmap of selected marker genes for the clusters shown in **k**. Rows are genes, columns are cells. Cells originating from clusters 1–4 are annotated with their respective colors in the boxes in the top. Color gradient depicts the expression *Z*-score relative to the average cell (unclustered). For the genes colored in blue or red to the left of the heatmap, a browser screenshot of their genomic locus is shown in Figure S2I. *Some of the panels in these figures contain public data. These panels are annotated with [PD]. The accession numbers of public data and their corresponding panels are annotated in* Additional file 2: Table S1

We next sought to use our STARR-seq approach to investigate known and novel differences in enhancer usage between 2iL and SL mouse ESCs and examined the C1 STARR-seq peaks (*n* = 18,544). Differential STARR-seq analysis using a model that accounts for differences in sequencing depth and input coverage (Additional file 1: Fig. S2A; methods) revealed 1442 enhancers that are significantly stronger in 2iL compared to 3688 in SL (fold change ≥ 2.5, *p* < 0.05, Fig. 2a; Additional file 4: Table S3). As expected, differential STARR-seq signal at C1-loci was in line with DNA accessibility, histone modification, and cofactor occupancy dynamics between 2iL and SL (Additional file 1: Fig. S2B). Motif analysis revealed enrichment for TCF/LEF, ZIC2/3, and ZFP281 motifs at 2iL- and SL STARR-seq peaks, respectively (Fig. 2b). TCF3 (*Tcf7l1*) is the highest expressed TCF/LEF member in ESCs, with similar expression in 2iL- and SL-ESCs (Additional file 1: Fig. S2E). Instead, de-repression of TCF/LEF activity is a well-known effect of CHIR99021-mediated GSK3-inhibition [51–53] that works through altered WNT-signaling, rather than modified *Tcf7l1* expression levels. Switching ESCs from SL to 2iL induces chromatin accessibility and H3K27ac at TCF3 occupied enhancers, as for example, near the “ground-state” pluripotency gene *Tcfcp2l1* (Fig. 2c). In SL, *Tcf3*^−/−^ ESCs also gain enhancer activity at sites that are otherwise repressed by TCF3 in STARR-seq, but do not reach the activity level as in 2iL-cultured cells (Additional file 1: Fig. S2C-D), in line with the notion that functional redundancy exists between TCF3 and other TCF/LEF factors [54].

### ZIC3 activates enhancers in SL-ESCs, but not in 2iL-ESCs

Having confirmed the known functional difference of the TCF/LEF family proteins between 2iL and SL ESCs, we next focused on the SL-induced STARR-seq peaks enriched for ZIC motifs. ZIC2 and ZIC3 are the two highest expressed ZIC genes in mESCs. ZIC3 expression is 3-fold higher in SL than 2iL, whereas ZIC2 expression is similar in 2iL and SL, but induced in EpiLCs (Additional file 1: Fig. S2E). Given these results and the pronounced difference in ZIC3 protein abundance between 2iL- and SL-ESCs (Fig. 2d, left), we focused on ZIC3. To determine whether ZIC3 indeed occupies STARR-seq peaks, we profiled ZIC3 occupancy by ChIP-seq in 2iL and SL and detected a total of ~ 11,800 peaks, mostly at distal sites. Twenty-seven percent (2590/8200) of the distal peaks had significant STARR-seq signal in 2iL- or SL-ESCs (Additional file 1: Fig. S2F). Of these, 927 ZIC3-bound sites had significantly higher STARR-seq signal in SL-ESCs (FC > 2.5, *p* < 0.05), but not a single ZIC3 binding site had higher STARR-seq signal in 2iL (Additional file 1: Fig. S2G). To assess whether ZIC3 contributes to enhancer activity, we depleted ZIC3 using CRISPR-CAS9 (Fig. 2d, right). *Zic3*^*−/−*^ mESCs appear morphologically normal when cultured in 2iL, but SL-ESCs displayed flattened colonies (Fig. 2e). Luciferase assays for selected enhancers in WT and *Zic3*^−/−^ ESCs showed that enhancer signal is lost upon genetic ablation of ZIC3 in SL-, but not in 2iL-ESCs, where the luciferase signal even increases compared to WT (Fig. 2f, g).

### *Zic3*^−/−^ SL-ESCs display increased differentiation towards the early endodermal cell fate

The altered cell morphology of *Zic3*^−/−^ SL-, but not 2iL-ESCs prompted us to determine the transcriptomes of *Zic3*^−/−^ and WT ESCs in the two culture conditions by RNA-seq. Principal component analysis (PCA) showed that genetic ablation of *Zic3* shifts the expression profile of cells cultured in SL, but barely affects that of cells cultured in 2iL (Fig. 2h). Differential expression analysis supported these results showing only 43 differentially expressed genes (DEGs) in 2iL (FC > 2.5, *p* < 0.05). In contrast, we detected 945 DEGs in *Zic3*^−/−^ cells cultured in SL compared to WT, of which 70% were upregulated in the Zic3 KO cells (Additional file 1: Fig. S2H; Additional file 5: Table S4). Marker genes of the primitive endoderm (PrE), like Sox7, *Sox17*, *Gata4*, *Cubn*, and *Dab2* [55] are all upregulated by 8-fold or more in SL (*p* < 2e−15 for all), in line with results reported for *Zic3* RNAi ECSs cultured in SL [56] (Fig. 2i). Interestingly, none of these genes are differentially expressed in 2iL-ESCs (data not shown). Simultaneously, ground-state pluripotency markers like *Nanog* (FC = − 2.4, *p* = 7.2 × 10^− 9^), *Klf2* (FC = − 4.85, *p* = 5.1 × 10^− 25^), *Tcfcp2l1* (FC = − 3.4, *p* = 5 × 10^− 18^), *Esrrb* (FC = − 2.4, *p* = 1.4 × 10^− 9^), and most prominently, *Prdm14* (FC = − 8.9, *p* = 7.2 × 10^− 14^) are significantly downregulated in *Zic3*^−/−^ SL-ESCs, although most of them are still highly expressed (Fig. 2j). Simultaneous retention of pluripotency gene expression and strong upregulation of PrE markers indicates that a fraction of *Zic3*^−/−^ cells differentiated towards the endodermal lineage. To test this hypothesis, we applied single cell RNA-seq. Cluster analysis on single-cell expression profiles identified four transcriptionally distinct clusters of cells (Fig. 2k). Cluster 1 consists of intermingled ZIC3 WT and ZIC3 KO ESCs cultured in 2iL. Cluster 2 is WT SL-ESCs and cluster 3 are *Zic3*^−/−^ ESCs cultured in SL. Downregulation of naïve (e.g., *Nanog, Prdm14*) and primed (e.g., *Lefty1*, *Skil*) pluripotency factors, combined with upregulation of DNA methyltransferases (DNMTs) discriminates cluster 2 from cluster 3. The fourth cluster comprising *Zic3*^−/−^ ESCs consists of cells cultured in SL that express high levels of the endodermal transcription factors and lost expression of pluripotency markers (Fig. 2l). *Nanog* and *Prdm14* are naïve pluripotency genes that lose expression upon deletion of *Zic3* in SL (Fig. 2l; Additional file 1: Fig. S2I, left). The primed pluripotency gene *Lefty1* and the neuronal marker *Gbx2* are among the top markers that discriminate *Zic3*^−/−^ cells from WT cells cultured in SL and are in the vicinity of ZIC3 occupied STARR-seq peaks with a significantly elevated signal in SL-ESCs (Fig. 2l; Additional file 1: Fig. S2I, right).

In summary, our STARR-seq data corroborates that TCF3 represses enhancers in SL, and to a much lower degree in 2iL. More striking is the significantly elevated STARR-seq signal at enhancers occupied by ZIC3 when ESCs are cultured in SL as compared to 2iL. Luciferase experiments show that enhancer activity is lost for *Zic3*^−/−^ ESCs cultured in SL, but gained in 2iL compared to WT cells. In line with these data, the transcriptional profile of *Zic3*^−/−^ ESCs cultured in SL shifts significantly compared to WT, with over 900 DEGs. In contrast, the RNA-seq profile of ESCs cultured in 2iL barely deviates from WT cells, suggesting that ZIC3 is neither an activator nor a repressor in 2iL-ESCs. A plausible explanation is the significantly higher expression of *Zic3* in SL compared to 2iL, which is even more striking at the protein level. Finally, 70% of the ~ 900 DEGs are upregulated in SL, with a remarkably high increase for endodermal marker genes. Single-cell RNA-seq corroborated and extended that a small fraction of SL-ESCs adapts a (primitive) endodermal fate, which explains the dramatic upregulation of these genes in our bulk RNA-seq.

### Class C2 STARR-seq loci are repressed by DNA methylation and H3K9me3

STARR-seq signal at C2-loci (Fig. 1d) shows that the TFs that boost transcription from the minimal promoter in the reporter assay are indeed expressed in ESCs but seemingly impaired to boost transcription in the chromatin context. The absence of chromatin accessibility and active histone marks at the C2-loci suggest that in ESCs, nucleosomes, DNA methylation, or repressive histone modifications prevent activation of these putative enhancers in the presence of chromatin. To test this hypothesis, we compared DNA methylation, H3K9me3, and H3K27me3 across the C1- to C3-loci and a set of randomly selected loci (methods). First, DNA methylation is significantly elevated at C2-loci compared to randomly selected loci, but depleted at C1- and C3-loci. Interestingly, this is even more apparent in the globally hypomethylated 2iL-ESCs [22] (Additional file 1: Fig. S3A).

**Fig. 3:**
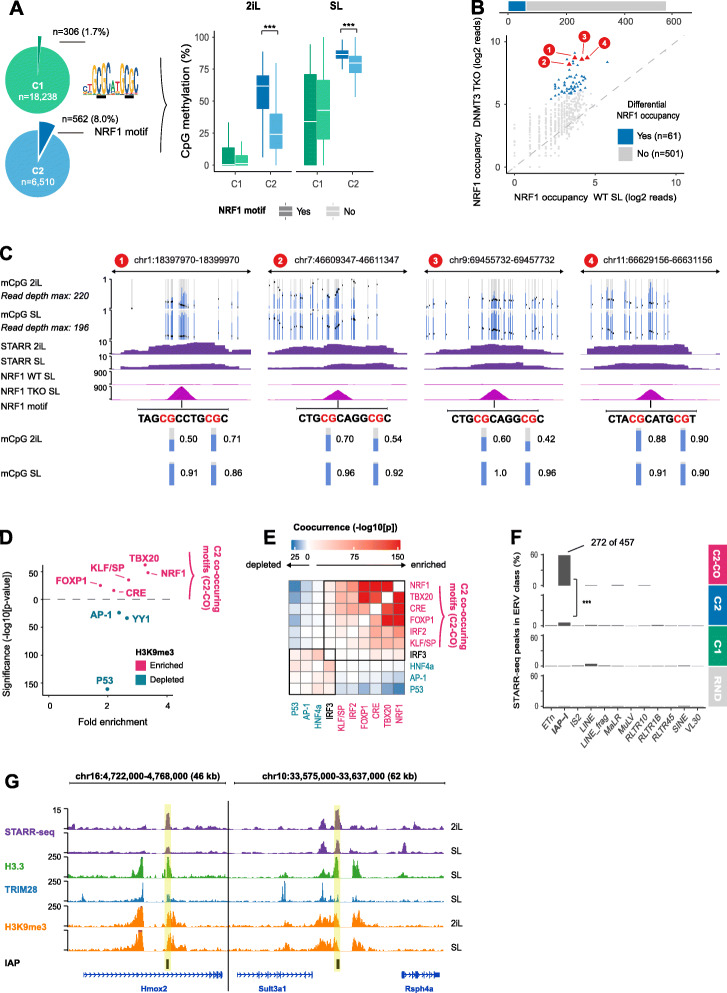
NRF1 and endogenous retroviruses establish active enhancers in the absence of DNA methylation or repressive histone modifications. **a** Left: Pie chart depicting the number of NRF1 motifs at C1 and C2 STARR-seq peaks. Right: Boxplots of CpG methylation at C1 and C2 STARR-seq peaks with (dark color) or without (light color) NRF1 motif. CpG methylation is significantly higher at C2 STARR-seq peaks that harbor a NRF1 motif, compared to those without a NRF1 motif. ***p < 2e−16, Wilcoxon rank-sum test [PD]. **b** NRF1 ChIP-seq occupancy at the *n* = 562 C2 STARR-peaks in WT and DNMT TKO ESCs cultured in SL. Top: Barplot that depicts that 61 of the 562 C2 STARR-peaks significantly gain NRF1 occupancy (*p* < 0.05, DESeq2, Wald test) in DNMT TKO cells. Bottom: Scatterplot that depict NRF1 occupancy in WT and DNMT3 TKO ESCs cultured in SL. NRF1-bound loci with differential occupancy are shown in blue. Four selected examples (red) are shown in **c** [PD]. **c** CpG methylation, STARR-seq, and NRF1 occupancy for the four example regions highlighted in **b**. Top: MethylC tracks [58] with blue bars depicting the fraction of methylated CpGs and black dots denoting the coverage depth. Purple: STARR-seq enrichment over input in 2iL and SL-ESCs. Middle: NRF1 occupancy in WT and DNMT TKO ESCs cultured in SL (RPKM). Bottom: NRF1 motif locations and CpG-methylation for the annotated CG-dinucleotides in 2iL and SL. [PD]. **d** DNA motifs enriched (pink) or depleted (blue) at C2 STARR-seq peaks that overlap a H3K9me3 peak relative to C2 STARR-seq peaks that do not overlap a H3K9me3 peak. Enriched motifs frequently co-occur (see **e**). **e** Co-occurrence of TF motifs enriched in **d**. *P* values were derived by the chi-squared test and corrected for testing multiple motif pairs (Benjamini-Hochberg). **f** STARR-seq peaks overlapping different ERV categories. The IAP-I ERV retroviruses overlap 60% of the C2-loci where the TBX20, NRF1, and KLF/SP (TNK) motifs co-occur. ***p < 2e−16, hypergeometric test. **g** Examples of IAPs with a STARR-seq peak (yellow highlight) covered by H3.3, TRIM28, and H3K9me3. STARR-seq tracks depict the enrichment over input, all other tracks depict RPKM [PD]. *Some of the panels in these figures contain public data. These panels are annotated with [PD]. The accession numbers of public data and their corresponding panels are annotated in* Additional file 2: Table S1

To gain insights into the TFs that activate these STARR-seq loci, we used linear regression and fitted their average DNA methylation percentage to TF motif presence. This analysis revealed that the presence of a P53 motif and the nuclear respiratory factor 1 (NRF1) motif are predictive of elevated DNA methylation (Additional file 1: Fig. S3B). These TFs are enriched in C2 enhancers that lack histone marking and accessibility (Fig. 1h). CpG-methylation has been shown to impede NRF1 occupancy [57] and C2-loci with a NRF1 motif have significantly higher methylated CpG levels than those without, which even applies to the globally hypomethylated 2iL-ESCs (Fig. 3a). Triple depletion of the *DNMT* genes causes global loss of CpG methylation (Additional file 1: Fig. S3C), but NFR1 occupancy is only significantly gained at a relatively small number of C2-loci harboring the NRF1 motif (Fig. 3b, c). This indicates that the sole loss of DNA methylation of its cis-acting element is insufficient to facilitate NRF1 binding and that alternative repressive mechanisms are at play. Hence, we examined for the presence of H3K9me3 and H3K27me3 in ChIP-seq data sets previously generated in our lab [10, 16]. H3K9me3 covers 21% of the C2-loci compared to 6% of C1-, 1% of the C3-, and 3% of the randomly selected loci (Additional file 1: Fig. S3D, F). H3K27me3 peaks overlap less than 0.1% of the C2-loci (Additional file 1: Fig. S3E-F). These results corroborate the EpiCSeg chromatin segmentation, where H3K27me3 peaks are mainly found together with “active” histone modifications and H3K9me3 covered loci are inaccessible, void of other histone modifications, and thus classified as C2-loci (Additional file 1: Fig. S1F).

### STARR-seq reveals endogenous retrovirus enhancers that are repressed by TRIM28

To investigate whether H3K9me3 is associated with specific TFs, we analyzed DNA motifs enriched at C2 STARR-seq peaks coinciding with H3K9me3 peaks as compared to C2-loci without H3K9me3. Motifs enriched and co-occurring at H3K9me3 repressed enhancers are TBX20, FOXP1, CRE, and also NRF1. In contrast, AP-1, YY1, and in particular P53 are found in loci without active marks that are also without H3K9me3 (Fig. 3d, e). Mining the Cistrome database [59, 60], we could not find evidence that these loci gain chromatin accessibility or TF occupancy in somatic cells (data not shown). Instead, these loci are hallmarked by the presence of the histone variant H3.3 and the repressor TRIM28 (KAP1) in ECSs, reminiscent of endogenous retroviruses (ERVs) [61, 62]. STARR-seq enrichment at H3K9me3 repressed ERVs has recently been reported in human HeLa-S3 cells [42]. To examine whether our C2-loci are also enriched at ERVs, we intersected them with a list of established retrotransposons [63]. This revealed that in particular the C2-loci that harbor at least three of the four TBX20, FOXP1, CRE, and NRF1 motifs are significantly enriched for intracisternal A-type particles (IAPs) (Fig. 3f; *p* < 2e−16; Fisher’s exact test). This result agrees with the finding that in particular IAPs gain expression upon TRIM28 deletion [62]. Browser examples of such TRIM28 repressed IAPs are shown in Fig. 3g.

In short, a significant portion of the C2 STARR-seq peaks are repressed by DNA methylation and H3K9me3, but display enhancer activity in our plasmid-born STARR-seq assay.

### P53 occupied enhancers have limited DNA accessibility and are split into two categories based on active histone modifications including H3K27ac

**Fig. 4:**
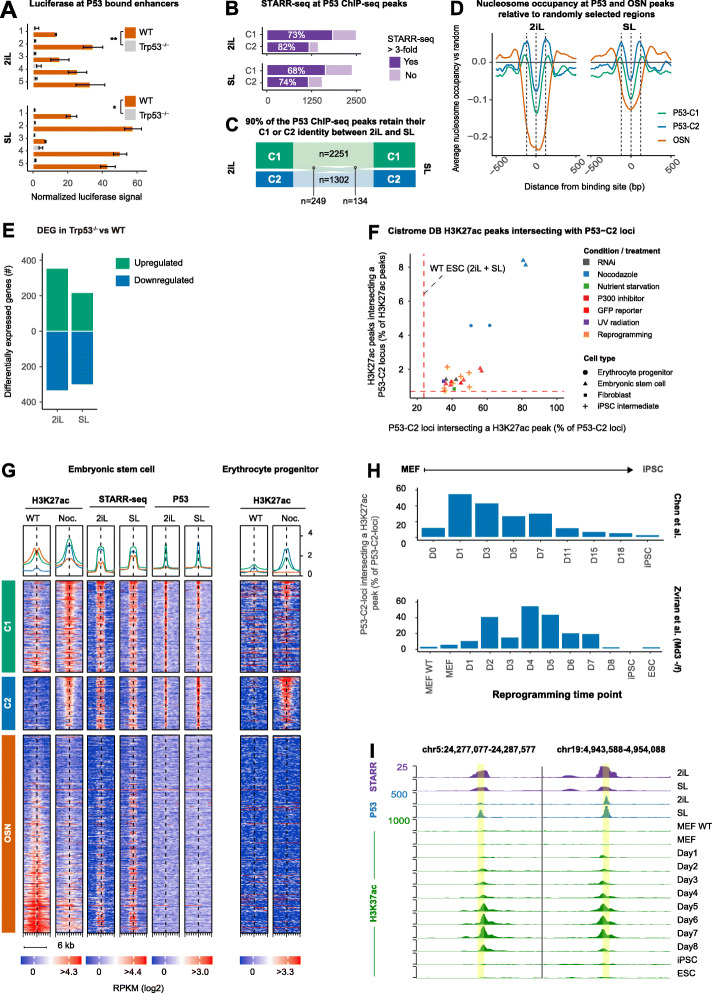
P53-occupied C2 enhancers gain H3K27ac upon treatment with Nocadazol and transiently during MEF to iPSC reprogramming. **a** Luciferase signal in WT and *Trp53*^−/−^ ESCs at selected P53-bound STARR-seq peaks. **p = 0.007, *p = 0.02, paired *t* test, *n* = 5. Error bars denote the standard error of the mean for technical triplicates. **b** Number of P53-C1 and P53-C2 ChIP-seq peaks with STARR-seq enrichment (FC ≥ 3 and *p* < 0.05 in both replicates). The percentage of P53-bound loci with STARR activity is similar between 2iL and SL. **c** Riverplot showing the number of P53-C1 and P53-C2 STARR-seq peaks. Ninety percent of these peaks do not change from C1 to C2 class between 2iL and SL. 249 peaks change from a C1 in 2iL to a C2 in SL and 134 peaks change from a C2 peak in 2iL to a C1 peak in SL. **d** Average ATAC-seq-derived nucleosome occupancy of P53-C1, P53-C2, and OC4T-SOX2-NANOG (OSN) peaks relative to randomly selected regions. P53-C1 and P53-C2 regions show a similar nucleosome occupancy pattern, but the lower average nucleosome occupancy of P53-C1 regions indicate that these loci are more frequently depleted of nucleosomes compared to P53-C2 regions. OSN bound regions are even more frequently depleted of nucleosomes. Nucleosome occupancy was computed with NucleoATAC for each nucleotide and smoothed using a running median over 11 adjacent nucleotides (see the “Methods” section) [PD]. **e** Number of differentially expressed genes (DEGs; *p* < 0.05 and |FC| ≥ 2.5) in *Trp53*^−/−^ ESCs compared to WT. **f** Mutual overlap between P53-C2 loci (*n* = 1551) and H3K27ac peaks for *n* = 2032 murine H3K27ac ChiP-seq libraries as deposited in the Cistrome database. The 23 H3K27ac libraries that had the highest overlap with our P53-C2 loci are shown. H3K27ac peaks in Nocodazole-treated ESCs overlap more than 80% of the P53-C2 loci. At the same time, the 1551 P53-C2 loci also overlap 8% of all the H3K27ac peaks detected in the Nocodazole-treated ESCs. This high mutual overlap indicates that Nocodazole treatment induces a specific deposition of H3K27ac at P53-C2 loci. The red dashed lines show that 24% of the P53-C2 loci intersect a H3K27ac peak in WT ESCs, which comprises 0.7% of the total number of H3K27ac peaks detected in WT ESCs (union 2iL and SL). See Table S6 for accession numbers and descriptions of these samples. **g** Left: Heatmap of H3K7ac, STARR-seq, and P53 occupancy in ESCs. Loci are ordered by difference (descending) in Nocodazole-treated (Noc) vs untreated ESCs. Right. Same loci showing H3K27ac in Nocodazole-treated and untreated erythrocyte progenitors [PD]. **h** Mouse embryonic fibroblasts (MEFs) that are being reprogrammed to iPSCs transiently gain H3K27ac at P53-C2 loci [PD]. **i** Examples of P53-C2 loci that transiently gain H3K27ac during iPSC reprogramming [PD]. *Some of the panels in these figures contain public data. These panels are annotated with [PD]. The accession numbers of public data and their corresponding panels are annotated in* Additional file 2: Table S1

Analysis of the chromatin marking and motif analysis of C2 STARR-seq peaks (lacking an active chromatin state) revealed a noticeable enrichment for the P53 motif in the absence of H3K9me3 (Fig. 3d). Note that the P53 motif is also highly enriched at C1 STARR-seq peaks that do exhibit active chromatin marks. To understand the role of P53 at these epigenetically distinct STARR-seq peaks, we first confirmed that P53 indeed drives the STARR-seq signal using luciferase transfections in *Trp53*^−/−^ and WT ESCs (Fig. 4a; Additional file 3: Table S2). Next, we assessed if and how P53 boosts transcription from genes interacting with the epigenetically distinct C1- and C2-loci. To this end, we performed P53 ChIP-seq and found that the union of 2iL- and SL-ESC peaks comprises ~ 4000 P53 binding sites. Reassuringly, most P53 binding sites show a significant STARR-seq signal (Fig. 4b). Furthermore, ~ 90% of the P53 ChIP-seq peaks retain their C1/C2 classification in 2iL- and SL-ESCs (henceforth named P53-C1 and P53-C2 respectively), indicating that ESC culture differences are not the main driver of the epigenetic difference (Fig. 4c). Given that we observed a similar loss of enhancer signal after genetic depletion of P53 in 2iL- and SL-ESCs (Fig. 4a), a similar fraction of P53-associated enhancers (Fig. 4b) and a largely unmodified epigenetic landscape (Fig. 4c), we performed further analysis on the union of P53 binding sites detected in 2iL- and SL-ESCs.

We examined chromatin accessibility and histone modifications at P53-C1 and P53-C2 ChIP-seq peaks and compared them to typical pluripotency enhancers co-bound by OCT4-SOX2-NANOG and with randomly selected loci. P53-C1 peaks are enriched for H3K4me1, H3K27ac, and RNA polymerase II (Additional file 1: Fig. S4A). These marks are largely absent at P53-C2 peaks. Interestingly, chromatin accessibility is low or even absent at both P53-bound classes (Additional file 1: Fig. S4A-B; Additional file 1: Fig. S4F). It has been shown that P53 has pioneering activity [64, 65] and is capable of binding nucleosomal DNA [66–68]. Hence, we performed nucleosome occupancy and phasing analysis using ATAC- [69] and MNAse-seq [70, 71] and observed strong nucleosome phasing at P53 peaks compared to OSN-bound or randomly selected loci (Fig. 4d, Additional file 1: Fig. S4C). Even with a relaxed cutoff, only 23% of the P53-C2 and 50% of the P53-C1 peaks overlap a nucleosome-free region compared to over 80% of the OSN-bound loci (Additional file 1: Fig. S4D).

To assess to what extent P53-C1 and P53-C2 peaks regulate gene expression in ESCs, we applied RNA-seq in *Trp53*^−/−^ and WT cells. P53 loss resulted in 888 differentially expressed genes compared to WT ESCs (fold change ≥ 2.5 and *p* < 0.05, see the “Methods” section, Fig. 4e, Additional file 6: Table S5). P53-C1 peaks are significantly closer to the TSS of P53 targets, here defined as genes that are differentially expressed in *Trp53*^−/−^ ESCs (Additional file 1: Fig. S4E).

### Cellular stress and iPSC reprogramming induce H3K27ac at P53-C2 loci

Thus far, we found little evidence for enhancer activity of P53-C2 enhancers except in the plasmid-borne STARR-seq assay. Therefore, we questioned whether and when these sites become active enhancers and scored thousands of murine H3K27ac ChIP-seq samples from the Cistrome database for their mutual overlap with P53-C2 loci. This analysis revealed that P53-C2 loci strongly gain H3K27ac when exposed to UV irradiation, serum starvation, and in particular Nocodazole; an antineoplastic agent that induces cell-cycle arrest at the G2/M phase [72] (Fig. 4f; Additional file 7: Table S6). Nocodazole also induces H3K27ac in erythrocyte progenitors [73], suggesting that the observed characteristics are not limited to ESCs (Fig. 4f, g; Additional file 1: Fig. S4G). Interestingly, P53-C2 loci also gain H3K27ac at intermediate fibroblast to iPSC reprogramming timepoints (Fig. 4f). This transient induction became even more apparent when we called H3K27ac peaks in two recent, independent iPSC reprogramming studies [74, 75] (Fig. 4h-i).

Taken together, P53 at C2-loci unveils STARR-seq peaks that are not or lowly accessible by ATAC-seq. Active enhancer marks like H3K27ac and RNA polymerase II are present at P53-C1 loci, but are lacking at P53-C2 loci under normal physiological conditions. P53-C2 loci gain H3K27ac upon treatment of ESC and erythrocyte progenitors with Nocodazole as well as during reprogramming of fibroblasts to iPSC cells suggesting that these loci become active enhancers.

## Discussion

**Fig. 5:**
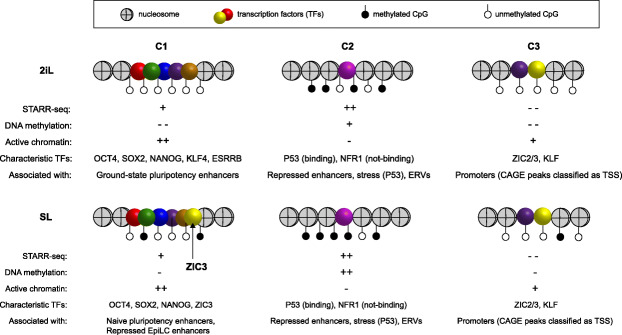
Schematic overview of the three enhancer classes and their main characteristics in 2iL- and SL-ESCs. C1 STARR-seq loci are accessible and covered by histone modifications associated with active enhancers. These loci have virtually no DNA methylation in 2iL- and low-DNA methylation in SL-ESCs. C1-loci are typically bound by well-known pluripotency factors like OCT4 (*Pou5f1*), SOX2, NANOG, and ESRRB in 2iL- and SL-ESCs. ZIC3 is a TF that is much more abundant in SL- compared to 2iL-ESCs and is associated with significantly stronger enhancer signals in SL-ESCs. C2 STARR-seq loci are not or very lowly covered by enhancer-associated histone modifications. C2-loci appear to be repressed in chromatin by high-DNA methylation and H3K9me3. In plasmid assays, the absence of these repressive marks causes NRF1-induced enhancer activity at a number of loci. Furthermore, enhancer associated with ERVs appear to be repressed by H3K9me3, but are active in our STARR-seq assays. A large group of C2-loci is associated with P53 motifs. P53 binding is not impeded by DNA methylation or H3K9me3 in WT ESCs, but the lack of enhancer-associated histone modifications indicate that these enhancers are silent under normal physiological conditions. A strong increase in H3K27ac after Nocodazole treatment and at intermediate-induced reprogramming timepoints shows that these enhancers can become activated in their endogenous context. C3-loci show low histone marking and accessibility associated with active regulatory elements, but no STARR-seq signal. We find C3-loci even at regions that were highly covered by our input DNA libraries. C3-loci further show very low CpG-methylation and overlap significantly with promoters classified by the Phantom5 consortium using CAGE analysis. This indicates that C3-loci largely overlap with promoters that are currently undefined in the Gencode or Refseq gene annotations. *Some of the panels in these figures contain public data. These panels are annotated with [PD]. The accession numbers of public data and their corresponding panels are annotated in* Additional file 2: Table S1

In this study, we used STARR-seq to generate a compendium of genomic loci with enhancer potential in murine ESCs. We found that 25% (6396 of 25,616) of our STARR-seq peaks overlap an ATAC-seq accessible region, bound by P300 and covered by H3K27ac (APK-peak). To examine the chromatin state of the remaining 75% of our STARR-seq peaks, we segmented the mouse genome using chromatin accessibility assays, histone modifications, and TF ChIP-seq collected for 2iL- and SL-ESCs. Using this segmentation, we separated STARR-seq peaks as “active” (so-called C1-loci) or inactive (C2-loci) in each of the culture conditions. Although we now found that 72% (18,544 of 25,616) of the STARR-seq peaks are embedded in “active” chromatin, the remaining 28% (*n* = 7072) are void of active marks. Finally, APK-peaks well covered by the STARR-seq input libraries, but lacking STARR-seq signal (*n* = 1950; C3-loci) were examined. C3-loci overlapping promoter regions of transcripts annotated by Refseq and Gencode were removed, but of the remaining C3-loci ~ 60% overlapped a CAGE peak classified as a TSS by the Phantom consortium. The properties of C1–C3 loci and the TFs associated with each class are summarized in Fig. 5. Note that we used an older version of the STARR-seq plasmid in which the bacterial origin of replication (ORI) functions as a (competitive) core promoter [42]. Transcripts initiated at the ORI may not reach the end of the luciferase gene or may not be accurately spliced, which would have resulted in an underestimation of the enhancer strength. In most mammalian cells, a second confounding effect is that DNA transfection induces a type I interferon response that gives rise to “false positive” enhancer elements [42]. Importantly, mESCs do not display this response [76, 77].

Next, we set out to examine the TFs that are associated with the three classes of regulatory elements we identified. C1-loci are enriched for pluripotency factors like OCT4, SOX2, NANOG, ESRRB, and KLF4 and typified by a chromatin signature associated with active enhancers. Importantly, differential STARR-seq signal at C1-loci reflects chromatin accessibility and histone modification dynamics between the two culture conditions. Our STARR-seq data was reproducible between biological replicates and validated by luciferase assays at selected loci. Thus, our compendium of STARR-seq peaks is robust allowing us to leverage the difference between the enhancer activity of genomic loci inferred from their chromatin state and that observed in a plasmid context.

To validate the results of the STARR-seq approach and to derive additional insights, we examined C1 STARR-seq peaks with the differential signal between 2iL- and SL-ESCs. Motif enrichment analysis pointed to an overrepresentation of TCF/LEF motifs in STARR-seq active loci in 2iL. This result corroborates our STARR-seq approach as TCF/LEF depression is a well-established feature of 2iL-ESCs [51, 53] associated with upregulation of naïve pluripotency factors [51, 78]. At STARR-seq peaks elevated in SL-ESCs, we detected ZIC2/3 motif enrichment. Of note, ZIC3 protein is much more abundant in SL- than 2iL-ESCs. To better understand the role of this TF in the two conditions, we genetically deleted *Zic3* and discovered an increasing number of differentiated cells in SL-, but not in 2iL-ESCs. This result agrees with a strongly reduced luciferase signal in *Zic3*^−/−^ ESCs that is observed only in SL ESCs. *Zic3*^*−/−*^ ESCs show 900 DEGs in SL, but only 43 in 2iL. Moreover, 70% of the DEGs in SL are upregulated which includes markers of the early endodermal lineage [56]. Our single-cell transcriptome profiling confirmed these findings, but showed that only a small fraction of ESCs differentiated. The vast majority of *Zic3*^−/−^ SL-ESCs retain the expression of key pluripotency markers, but are distinct from their WT counterparts for example by upregulation of *Dnmt* genes. This increased propensity of *Zic3*^−/−^ ESCs to differentiate towards the endodermal linage has been shown before for cells cultured in SL [56] and could be associated with the diminished expression of key pluripotency genes like *Nanog* and *Prdm14*, repressors of the (extraembryonic) endodermal lineage [56, 79]. *Zic3* has also been shown to interact with primed pluripotency genes like *Otx2* and *Oct4* [31] and was recently identified as a key driver of the transition from naïve to primed pluripotency [80]. Loss of *Zic3* may therefore block the transition towards epiblast-like cells, resulting in cells that adopt an early endodermal cell fate when their pluripotency network is compromised.

A second class of STARR-seq peaks (class C2) have a chromatin signature associated with gene repression. These loci have higher DNA methylation and/or H3K9me3 occupancy compared to actively marked loci. Specifically, we found a significant STARR-seq signal at the CpG-rich binding sites of NRF1, a TF whose occupancy is restricted by DNA methylation [57]. Genetic deletion of the three DNMT genes causes global hypomethylation, but increases binding significantly at only 15% of the loci with a NRF1 DNA motif. Other STARR-seq peaks of the C2 class overlap IAP retrotransposons and are repressed by H3K9me3. The most striking C2-loci are those bound by P53. ChIP-seq showed that P53 does occupy C2 STARR-seq peaks, even though the loci displayed no or minimal ATAC/DNAse1 signal. On the other hand, P53 in the C1 class also occupies many STARR-seq peaks that do have active enhancer marks. These enhancers, although carrying active marks, also have strikingly low chromatin accessibility compared to promoters or typical pluripotency enhancers. P53 binds C2-labeled STARR-seq peaks in multiple tissues, and these loci gain significant amounts of H3K27ac when exposed to UV-irradiation, Nocodazole treatment, and interestingly also displayed transient increase of H3K27ac during reprogramming from fibroblasts to iPSCs. Further understanding this mechanism during iPSC reprogramming and its target genes will be of interest, as P53 is known to hamper reprogramming efficiency.

In conclusion, our STARR-seq assay revealed enhancers that recapitulate known differences between 2iL- and SL-ESCs. Active enhancers are accessible, marked with active histone modifications, and bound by pluripotency- and other known transcription factors. STARR-seq shows that TCF3 represses enhancers and that ZIC3 act as an activator in SL, but not in 2iL. Genetic loss of *Zic3* increases the fraction of SL-cultured cells that differentiate and remarkably, these cells adopt an endodermal cell fate. Finally, STARR-seq identifies chromatin-masked enhancers at which TF occupancy is impeded by repressive marks as well as dormant enhancers that are occupied by P53, but only gain H3K27ac in specific conditions. Our compendium of STARR-seq enhancers will be beneficial for future stem cell research, and we expect that a similar comparison between STARR-seq and chromatin-based assays will yield important regulatory insights in other cell types.

## Methods

### Experimental procedures

#### Cell culture

Mouse embryonic stem cells E14Tg2a (E14) were purchased from ATCC and cultured without feeders in the presence of LIF. SL-ESCs were maintained in DMEM with 15% ES grade fetal calf/bovine serum. 2iL-ESCs were maintained in Ndiff227 (Takara Bio, Inc.) medium supplemented with 1-μM MEK inhibitor PD0325901 and 3-μM GSK inhibitor CHIR99021. Zic3 KO and Trp53 KO ESCs were tested by western blot. E14 cells were purchased from ATCC and were not authenticated.

#### Construction of the STARR-seq plasmid pool

The mouse genome was isolated and sonicated. Sheared genomic DNA fragments of 700 ~ 1200 bp were size selected on 1% agarose gel to construct the STARR-seq plasmid pool following the STARR-seq protocol [38]. Illumina adapters using NEBNext® Ultra™ II DNA Library Prep Kit for Illumina (NEB, #E7645S) were added to the size-selected DNA fragments. Adapter-ligated fragments were amplified with library cloning primers [38], which added a 15 nt sequence to the adapters for ligation. Totally, 5 μg fragmented genome DNA was provided with adapters and amplified for 5 cycles in 10 reactions. The PCR products were purified with 0.8x AMpure XP beads (Agencourt, #A63882) and then ligated to Age1/Sal1-digested hSTARR-seq_SCP1 vector (Addgene, #99292) following the instruction of In-Fusion HD Kit (Clonetech #639649). The ligation products were purified and transformed into MegaX DH10B™ T1R Electrocomp™ Cells (Invitrogen, #C640003). Plasmid Plus Giga Kit (Qiagen, #12991) was used to extract the plasmid pool for screening.

#### Preparation of STARR-seq libraries

Four hundred micrograms of plasmid pool was transfected into 400 million cells with Lipo3000 (Invitrogen; L3000015). The medium was refreshed after 12 h and cells harvested after 24 h. We isolated total RNA with the RNeasy maxi prep kit (Qiagen, #75162) from half of the harvested cells (~ 0.2 billion cells) and incubated the total RNA with Dynabeads Oligo-dT25 (Invitrogen, #61002) to isolate polyA+ RNA. The polyA+ RNA was concentrated following the clean-up procedure of RNeasy Mini kit (Qiagen, #74106) and was transcribed in 24 reactions with reporter-RNA specific primer and cDNA amplified with reporter-specific primers for 15 cycles [38]. The amplified cDNA was purified with 0.8x AMpureXP beads. Illumina indexes for sequencing were added to the amplified cDNA according to PCR for 8 cycles with NEBNext Multiplex Oligos for Illumina (NEB, #E7335L and #E7500L) in 24 reactions. The indexed libraries were size-selected on 1% agarose gel to remove the remaining small fragments.

The reporter constructs were isolated from the second half of the transfected cells with Plasmid Maxi Kit (Qiagen, #12165) as input. Every 100 ng input was amplified using input-specific primers (GATTTGATATTCACCTGGCCCGC & CTTATCATGTCTGCTCGA*A*G*C, where * indicates phosphorothioate bonds) and then size-selected (1600 bp ~ 2200 bp) on 1% agarose gel in 10 reactions. The indexes were added to purified input DNA following the same indexing procedure with STARR cDNA in 10 reactions and purified on 1% agarose gel.

#### ChIP-seq

Cells were fixed with 1% formaldehyde for 10 min followed by 0.125 M glycine to stop the reaction. The fixed cells were washed with PBS twice and then scraped and collected into 50 ml falcon tubes. Every 10 million cells were pelleted and snap-frozen in liquid nitrogen and then stored in − 80 freezer for ChIP. The cells were lysed in 300 μl 1% SDS buffer (50 mM Tris-HCl pH 8.0, 1% SDS, 100 mM NaCl, 5 mM EDTA, protease, and phosphatase inhibitors) and sonicated to 200 bp ~ 600 bp. Three hundred microliters of fragmented chromatin was diluted with 3 ml IP buffer (0.01% SDS,1% Triton, 2 mM EDTA, 50 mM Tris-HCl, 150 mM NaCl, protease inhibitor cocktail) and pre-cleared with Protein A/G beads for 1 h on a rotating wheel at 4 °C. The used beads were trashed and the pre-cleared chromatin was incubated with antibody and unused protein A/G beads overnight at a 4 °C rotator. Beads were washed successively in 4 °C with low salt washing buffer (0.1%SDS, 1% Triton-100, 2 mM EDTA, 20 mM Tris-HCl pH 8.0, 150 mM NaCl), high salt washing buffer (0.1%SDS, 1% Triton-100, 2 mM EDTA, 20 mM Tris-HCl pH 8.0, 500 mM NaCl), LiCl washing buffer (0.25 M LiCl, 1% NP-40, 1% deoxycholate, 1 mM EDTA, 10 mM Tris-HCl pH 8.0), and TE buffer (1 mM EDTA, 10 mM Tris-HCl pH 8.0). Beads were transferred to new Eppendorf tubes and washed one more time with TE buffer. Washed beads were resuspended in 200 μl elution buffer (1% SDS, 0.5 mM EDTA, 50 mM Tris-HCl pH 8.0), and then, the tubes were placed in the thermo mixer at 65 °C and 1000 rpm for 30 min. Supernatants were transferred to new tubes that contained 8 ul 5 M NaCl and were de-crosslinked overnight in a thermo mixer at 65 °C. DNA was purified with MinElute PCR Purification Kit (Qiagen, #28006) following treatment with RNase and Proteinase K. Libraries for sequencing were constructed following the KAPA Hyper Prep Kit (Kapa Biosystems, #KK8504). The antibodies used in this study were P53 (Novocastra laboratories #NCL-p53-CM5p) and ZIC3 (Abcam #222124).

#### RNA-seq

RNA was extracted using the RNeasy Mini Kit (Qiagen #74106). One microgram of RNA was used to construct the library using the KAPA RNA HyperPrep Kit with RiboErase (KAPABiosystems #KK8560).

#### scRNA-seq

For scRNA-seq, cells were processed using SORT-seq (CEL-seq2-based scRNA-seq; cells sorted into 384-well plates [81]). Cells were trypsinized and sorted into 384-well plates. The cells were lysed at 65 °C for 5 min and then processed with first- and second-strand RT reaction. The aqueous phase was separated from the oil phase after pooling the contents of all wells, followed by IVT transcription (Invitrogen # AM1334). The RNA was fragmented and cleaned up with AMpureXP beads. Half of the concentrated RNA was used for library preparation following the CEL-seq2 protocol [82]. The other half was stored at − 80 °C as a backup.

#### CRISPR-Cas9 genome editing

Nickase Cas9n was used to delete the target genome regions. The vector was purchased from Addgene (pSpCas9n(BB)-2A-Puro (PX462) V2.0, #62987). Online tools (crispr.mit.edu and Benchling) were used to design guide RNAs that were cloned into the vector (Table S3). mESCs were transfected with 2 μg cloned plasmid pool. Single colonies were picked after 2-day treatment with 1.5 μg/ml puromycin (InvivoGen #ant-pr-1) and amplified. Genomic DNA from individual colonies was extracted as a template for PCR amplification and Sanger sequenced to identify the genome pattern of the edited colonies.

#### Dual-luciferase assay

We replaced the PGL4.24 mini-promoter with the SCP1 promoter used in STARR-seq. Fragments to be tested (Table S3) were amplified and inserted into the backbone downstream of the luciferase. One hundred twenty nanograms of reporter plasmid and 5 ng TK-promoter Renilla plasmid were co-transfected into 50,000 cells in 1 well of a 96-wells plate. Candidate fragments were tested in at least three technical replicates. The medium was replaced after 12 h, washed with PBS twice, and lysed after 24 h. The assay was performed following the manufacturer’s instruction of Dual-Luciferase® Reporter Assay System (Promega, #E1910). The samples were mixed with LARII and Stop&Glo and measured in luminometer (Perkin Elmer 1420 Victor3).

#### Immunoblotting

Cells were trypsinized and then washed with 1x PBS twice. The pellet was lysed in RIPA buffer (150 nM NaCl, 1% NP-40, 0.5% NaDOC, 0.1%SDS, 50 mM Tris-HCl pH 8.0) with fresh added EDTA-free protease inhibitor cocktail (Roche #4693132001). Protein concentration was measured with the Bio-rad protein assay (Bio-Rad #500-0006). Cell extracts were loaded and separated by 10% SDS-PAGE gel, electrotransferred to nitrocellulose membranes and incubated with primary antibody (1:1000 diluted in blocking buffer) overnight at 4 °C, and then washed with 1x TBST for 5 min 5 times. Next, the membrane was incubated with a second antibody at room temperature for 1 h and then washed. ECL substrate (Thermo #32106) was added and images were acquired. The primary antibodies used in the study are ZIC3 (Abcam #222124) and GAPDH (Abcam #8245). The secondary antibodies used are Swine anti-Rabbit HRP (Dako #P0217) and Rabbit anti-Mouse HRP (Dako #P0161).

### Data analysis

#### STARR-seq library mapping and quality control

STARR-seq libraries were sequenced on an Illumina Nextseq 500. Paired-end FASTQ files were mapped with *BWA MEM* [83] using standard settings. Low-quality (MAPQ < 10) reads and non-properly paired reads were discarded. Library complexity (*EstimateLibraryComplexity*), GC% bias (*CollectGcBiasMetrics*), insert size metrics (*CollectInsertSizeMetrics*), and PCR duplication (*MarkDuplicates*) were estimated with *PICARD Tools.*^1^ Genome coverage was computed with *bedtools* (v2.27.1) [84] using the function “genomecov.”

#### STARR-seq peak calling and enrichment

Initial STARR-seq peaks were called for each library with *MACS2* [45] using the library matched transfected input libraries as control with parameters “keep-dup = all,”, “bandwidth = 800,” and “*p* = 1e−8.” The number of input reads, unique (non-duplicated) STARR-seq reads, and total STARR-seq reads within each library were counted with *featureCounts* [85]. Input read counts were normalized to the STARR-seq libraries and ceiled to the nearest integer. STARR-seq significance per peak was computed using a right-tailed binomial model applied to each library, with:

- # successes = total number of STARR-seq reads per peak;
- # trials = max (input reads per peak, unique STARR-seq reads per peak), using the rationale that each unique STARR-seq fragment must have been in the input library;
- Probability of success = 0.5 (after library scaling half of the reads are input).

To determine a suitable enrichment- and FDR threshold, 1 million GC% matched random sequences with median length matching the STARR-seq peaks were sampled per library. Binomial *p* values were corrected for multiple testing using the Benjamini-Hochberg correction [86]. Enrichment values (# successes/# trials) at STARR-seq peaks and random regions were shrunken using Bayesian shrinkage. Briefly, a beta-binomial model was fitted to the STARR-seq peaks and shrinkage parameters α and ß were optimized using maximum likelihood estimation, implemented in the *VGAM* R-package [87]. A 3-fold enrichment over input was observed in less than 3% of the randomly sampled regions (empirical FDR < 0.03). Therefore, our final set of STARR-seq peaks have an enrichment ≥ 3 per culture condition (merged replicates) and adjusted binomial *p* value < 0.05 in both individual libraries.

#### STARR-seq sequencing depth convergence analysis

To estimate the relation between the number of significantly enriched enhancers and library sequencing depth we first merged the two 2iL and SL libraries. Next, we downsampled the BAM files to a fraction (0.2, 0.3, …,0.9) of the original size and repeated the enhancer calling procedure above.

#### “Merging” of 2iL- and SL STARR-seq peaks

The union of the STARR-seq peaks called in 2iL- and SL (enrichment ≥ 3 and binomial *p* value < 0.05) was generated by concatenating the 2iL and SL peak BED files, followed by the “bedtools merge” command on the concatenated peak list. This “merged” list of 25,616 loci was used as follows:

- Identify genomic loci with STARR-seq activity in 2iL or SL;
- Use these genomic loci to compute differential STARR-seq activity between 2iL- and SL.

#### STARR-seq differential analysis

*DESeq2* [88] was used to compute differential STARR-seq signal within the “merged” STARR-seq peak set defined above. As above, the number of input reads, unique (non-duplicated) STARR-seq reads, and total STARR-seq reads were computed for each peak and within each library using *featureCounts*. Custom “normalizationFactors” were used to correct for STARR-seq and input sequencing depth, as well as input coverage at individual peaks. Specifically, the normalization factor *NF_ij* for STARR-seq peak *i* in library *j* is defined as follows:

NF_ij ≤ sizeFactor (STARR_j)/sizeFactor(input_j) * input_ij,

where the sizeFactor function is defined in the *DEseq2* paper [88] and implemented in the *DESeq2* R-paclage. *Input_ij* is the maximum of the input reads and the unique (non-duplicated) STARR-seq reads, using the rationale that a fragment cannot give STARR-seq signal if it was not in the input library. Finally, *NF_j* is scaled over the four libraries to have geometric mean = 1 following *DESeq2* recommendation. STARR-seq peaks with an absolute fold change ≥ 2.5 and *p* < 0.05 between SL and 2iL were considered differential.

#### Luciferase analysis

Luciferase signal was computed as the Firefly vs Renilla (FvR) signal averaged over 3 technical replicates, normalized by the FvR at a control region. Two batches of experiments were performed and batch-corrected using linear regression. To compare luciferase signal with STARR-seq, we log_2_ transformed the FvR values and linearly scaled the *n* = 39 values to the STARR-seq log_2_ enrichment values.

#### ATAC- and ChIP-seq analysis

ATAC-seq, ChIP-seq libraries, and public data were mapped using *Bowtie2* [89]. In the case of biological replicates, initial peaks were called using *MACS2* [45] in narrow peak mode with a *p* value threshold of 0.1, followed by *IDR* [90] with a threshold of 0.03. In the absence of biological replicates, peaks were called with *MACS2* using a *p* value threshold of 1e−8. For H3K27me3 and H3K9me3, we used *MACS2* in broad peak calling mode with parameters *p* = 1e−12 and broad-cutoff = 1e−5, based on a manual inspection in the IGV browser. Similarly, H3K27ac peaks in iPSCs were called using *MACS2* in broad peak calling mode with parameters *p* = 1e−15 and broad-cutoff = 1e−7. In all cases, *MACS2* was used with input DNA as control. Similar to the STARR-seq loci, *bedtools merge* [84] was used to take the union of 2iL and SL peaks, which served as a reference set for differential analysis. Reads within peaks were counted per library using *featureCounts* [85], followed by library normalization and differential analysis in *DESeq*2 [88]. Bigwig browser tracks were created using *deeptools bamCoverage* [91] and normalized to RPKM.

#### Nucleosome occupancy analysis

Nucleosome occupancy profiles of P53-bound and control loci were derived from ATAC-seq and MNAse-seq. In brief, our ATAC-seq libraries were merged with public ATAC-seq data of 2iL- and SL-ESCs to reach a better coverage (see Table S2). Nucleosome occupancy was averaged per bp and normalized to randomly selected occupancy for a region of 500 bp flanking the P53-motif (P53-loci) or peak center (OSN-bound and random loci). Nucleosome free regions were called using *NucleoATAC* [69] with the defaults setting: “–max_occ 0.1” or a relaxed cutoff: “–max_occ 0.2” and overlapped with the peak summit (1 bp region) of P53-C1, P53-C2, OSN, and random regions. Nucleosome occupancy was also analyzed with *Danpos2* [71] using MNAse-seq from Voong et al. [70] (GSM2183911). For each peak, the highest *Danpos2* nucleosome summit value within 74 bp from a peak summit was used.

#### Heatmap visualization

Heatmaps were made with the *EnrichedHeatmap* package for R [92]. Reads were counted using *featureCounts* [85] in bins of 50 bp flanking the feature of interest by 3 kb on both sides. Reads were normalized to RPKM, log_2_ transformed, and smoothed using a running median of 5 bins. The final values were capped between 75% and 90% of the maximum intensity to enhance visualization.

#### TF motif analysis

*Homer2* [93] was used to compute statistically enriched motifs in C1-, C2- or C3-loci relative to a background set. For C1- and C2-loci, motifs were searched in a region that flanks the STARR-seq summit by 250 bp. For C3-loci, motifs were searched at the peak center ± 250 bp. The background set consisted of randomly selected regions of 500 bp with at least 10 reads in each STARR-seq input library and a CG% distribution matching the C1–C3 STARR-seq peaks.

#### Cistrome analysis

BED files of P53 binding sites were generated using the location of the canonical P53 motif flanked by 250 bp if detected, or the peak summit flanked by 250 bp otherwise. BED files were converted to the mm10 reference genome using the UCSC liftover tools. Cistrome peaks called for murine H3K27ac libraries (BED files) were bulk-downloaded on April 30, 2019. Finally, peak sets were intersected using the R/Bioconductor package *GenomicRanges* [94].

#### RNA-seq analysis

RNA-seq libraries were mapped to the Gencode M1-NCBIM37 (mm9) reference genome using *STAR* [95]. Read normalization and differential analysis were performed using *DESeq2* with standard settings.

#### scRNA-seq analysis

Single-cell RNA-seq reads were first mapped to the Gencode M1-NCBIM37 (mm9) reference genome using *STAR*. UMI counts for a whitelist of 384- cell barcodes were determined using a custom R-script. Read normalization and cluster analysis were performed with the *Seurat* v3.0 R-package [96].

#### DNA methylation analysis

Whole genome bisulfite data for 2iL and SL-ESCs previously generated in our lab [22] were processed using *Bismark* [97]. The fraction mCpG was computed at C1, C2, C3, and random peak summits flanked by 250 bp on both sides.

## Supplementary information

**Additional file 1: Figure S1**. Quality assessment and chromatin-based classification of STARR-seq (lacking) loci. **Figure S2**. TCF3 and ZIC3 are associated with STARR-seq peaks induced in 2iL- and SLESCs respectively. **Figure S3**. C2-loci are active STARR-seq regions due to the absence of DNA methylation and repressive histone modifications. **Figure S4**. P53-bound STARR-seq loci are characterized by low chromatin accessibility and are categorized into active (class C1) and inactive enhancers (class C2) based on the presence/absence of active histone modifications.

**Additional file 2: Table S1**. Public data.

**Additional file 3: Table S2**. Primers and loci.

**Additional file 4: Table S3**. STARRseq 2iL SL ESCs.

**Additional file 5: Table S4**. Zic3 WT KO RNAseq.

**Additional file 6: Table S5** Trp53 WT KO RNAseq.

**Additional file 7: Table S6**. Cistrome ChIPseq enrichment.

**Additional file 8.** Review history.

## Data Availability

Raw and processed sequencing data have been deposited in Gene Expression Omnibus under accession GSE143546 [98]. The R-code and processed data to reproduce all figures in the manuscript are available at https://github.com/wmegchel/starrseq2020 [99] under the Apache 2.0 open source license. An archived copy of the version used in the publication is available at 10.5281/zenodo.3988187 [100] Additional file 2: Table S1 contains the accession numbers for all public data used in this study. Additional file 3: Table S2 contains the primer sequences and genomic loci for luciferase validation experiments and CRISPR-KO of Zic3. Additional file 4: Table S3 lists the (differential) STARR-seq loci. Genes differentially expressed in *Zic3*^−/−^ ESCs or *Trp53*^−/−^ ESCs are listed in Additional file 5: Table S4 and Additional file 6: Table S5 respectively. Additional file 7: Table S6 contains the 23 Cistrome H3K27ac datasets for which H3K27ac peaks had a higher overlap with the P53-C2 loci compared to WT ESCs. Additional file 7: Review history.

## Footnotes

1 http://broadinstitute.github.io/picard/
