# Supplementary material for: STARR-seq identifies active, chromatin-masked, and dormant enhancers in pluripotent mouse embryonic stem cells
Source: Genome Biol. 2020 Sep 10;21:243. doi: 10.1186/s13059-020-02156-3 (PMC7488044; doi:10.1186/s13059-020-02156-3)
Supplement: Supplementary file 1 — Additional file 1: Figure S1. Quality assessment and chromatin-based classification of STARR-seq (lacking) loci. Figure S2. TCF3 and ZIC3 are associated with STARR-seq peaks induced in 2iL- and SLESCs respectively. Figure S3. C2-loci are active STARR-seq regions due to the absence of DNA methylation and repressive histone modifications. Figure S4. P53-bound STARR-seq loci are characterized by low chromatin accessibility and are categorized into active (class C1) and inactive enhancers (class C2) based on the presence/absence of active histone modifications. [file 13059_2020_2156_MOESM1_ESM.pdf]

Figure S1

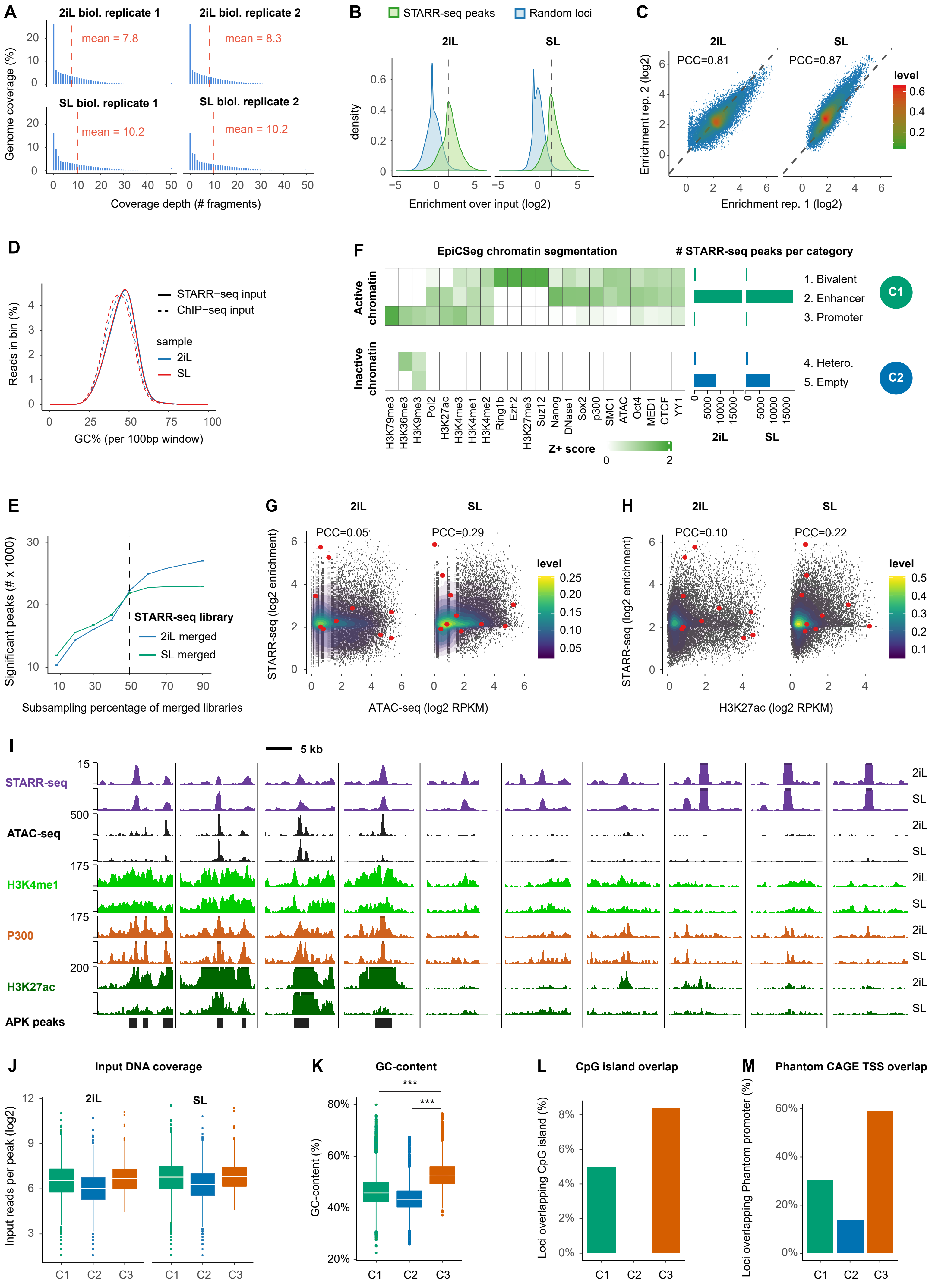
A
2iL biol. replicate 1
mean = 7.8
2iL biol. replicate 2
mean = 8.3
SL biol. replicate 1
mean = 10.2
SL biol. replicate 2
mean = 10.2
Genome coverage (%)
Coverage depth (# fragments)
B
STARR-seq peaks
Random loci
2iL
SL
density
Enrichment over input (log2)
C
2iL
SL
PCC=0.81
PCC=0.87
level
Enrichment rep. 2 (log2)
Enrichment rep. 1 (log2)
D
STARR−seq input
ChIP−seq input
sample
2iL
SL
Reads in bin (%)
GC% (per 100bp window)
F
EpiCSeg chromatin segmentation
STARR-seq peaks per category
Active chromatin
Inactive chromatin
1. Bivalent
2. Enhancer
3. Promoter
4. Hetero.
5. Empty
C1
C2
H3K79me3
H3K36me3
H3K9me3
Pol2
H3K27ac
H3K4me3
H3K4me1
H3K4me2
Ring1b
Ezh2
H3K27me3
Suz12
Nanog
DNase1
Sox2
p300
SMC1
ATAC
Oct4
MED1
CTCF
YY1
Z+ score
2iL
SL
E
Significant peaks (# x 1000)
STARR-seq library
2iL merged
SL merged
Subsampling percentage of merged libraries
G
2iL
SL
PCC=0.05
PCC=0.29
level
STARR-seq (log2 enrichment)
ATAC-seq (log2 RPKM)
H
2iL
SL
PCC=0.10
PCC=0.22
level
STARR-seq (log2 enrichment)
H3K27ac (log2 RPKM)
I
5 kb
STARR-seq
ATAC-seq
H3K4me1
P300
H3K27ac
APK peaks
2iL
SL
J
Input DNA coverage
2iL
SL
Input reads per peak (log2)
C1
C2
C3
K
GC-content
***
***
GC-content (%)
C1
C2
C3
L
CpG island overlap
Loci overlapping CpG island (%)
C1
C2
C3
M
Phantom CAGE TSS overlap
Loci overlapping Phantom promoter (%)
C1
C2
C3

Figure S2

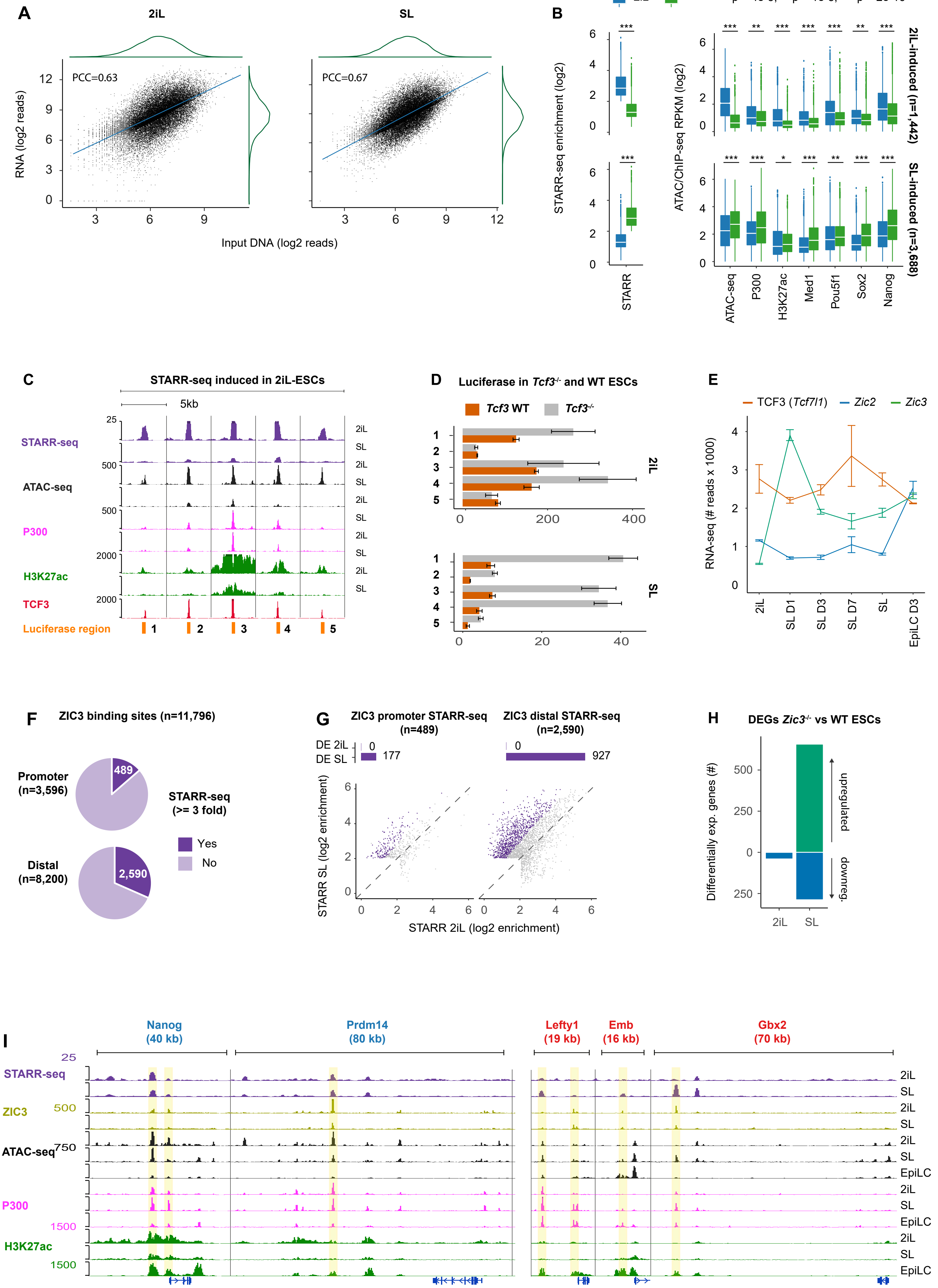
A
2iL
SL
PCC=0.63
PCC=0.67
RNA (log2 reads)
Input DNA (log2 reads)
B
2iL
SL
* p < 4e-5, ** p < 1e-8, *** p < 2e-16
STARR-seq enrichment (log2)
ATAC/ChIP-seq RPKM (log2)
STARR
ATAC-seq
P300
H3K27ac
Med1
Pou5f1
Sox2
Nanog
2iL-induced (n=1,442)
SL-induced (n=3,688)
C
STARR-seq induced in 2iL-ESCs
5kb
STARR-seq
ATAC-seq
P300
H3K27ac
TCF3
Luciferase region
D
Luciferase in Tcf3-/- and WT ESCs
Tcf3 WT
Tcf3-/-
E
TCF3 (Tcf7l1)
Zic2
Zic3
RNA-seq (# reads x 1000)
2iL
SL D1
SL D3
SL D7
SL
EpiLC D3
F
ZIC3 binding sites (n=11,796)
Promoter (n=3,596)
Distal (n=8,200)
489
2,590
STARR-seq (>= 3 fold)
Yes
No
G
ZIC3 promoter STARR-seq (n=489)
ZIC3 distal STARR-seq (n=2,590)
DE 2iL
DE SL
0
177
0
927
STARR SL (log2 enrichment)
STARR 2iL (log2 enrichment)
H
DEGs Zic3-/- vs WT ESCs
Differentially exp. genes (#)
upregulated
downreg.
2iL
SL
I
Nanog (40 kb)
Prdm14 (80 kb)
Lefty1 (19 kb)
Emb (16 kb)
Gbx2 (70 kb)
STARR-seq
ZIC3
ATAC-seq
P300
H3K27ac
2iL
SL
EpiLC

Figure S3

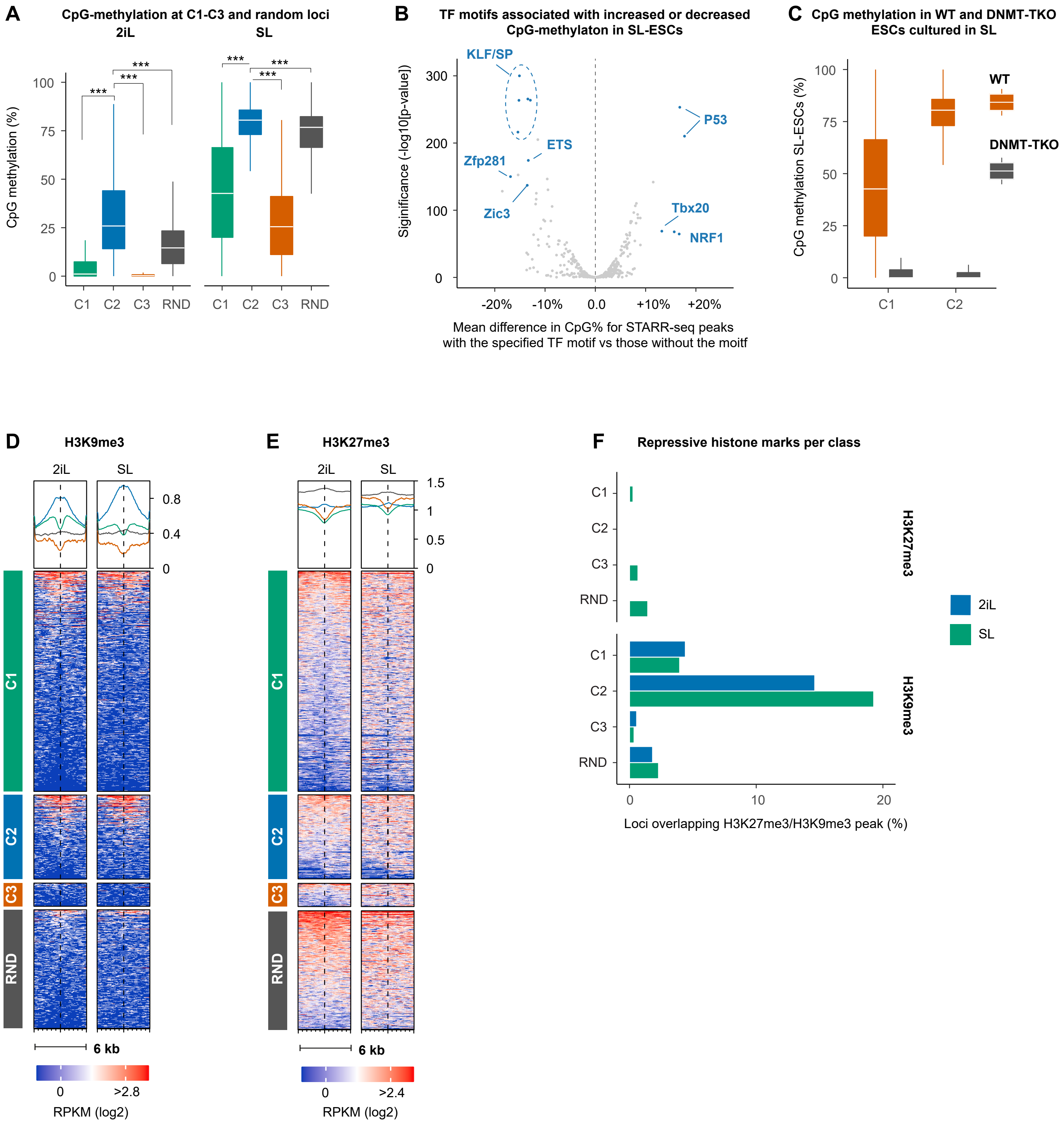
A
CpG-methylation at C1-C3 and random loci
2iL
SL
CpG methylation (%)
***
C1 C2 C3 RND C1 C2 C3 RND
B
TF motifs associated with increased or decreased CpG-methylaton in SL-ESCs
Siginificance (-log10[p-value])
KLF/SP
ETS
Zfp281
Zic3
P53
Tbx20
NRF1
-20% -10% 0.0 +10% +20%
Mean difference in CpG% for STARR-seq peaks with the specified TF motif vs those without the moitf
C
CpG methylation in WT and DNMT-TKO ESCs cultured in SL
CpG methylation SL-ESCs (%)
WT
DNMT-TKO
C1 C2
D
H3K9me3
2iL SL
C1 C2 C3 RND
6 kb
0 >2.8
RPKM (log2)
E
H3K27me3
2iL SL
6 kb
0 >2.4
RPKM (log2)
F
Repressive histone marks per class
H3K27me3
H3K9me3
2iL
SL
Loci overlapping H3K27me3/H3K9me3 peak (%)

Figure S4

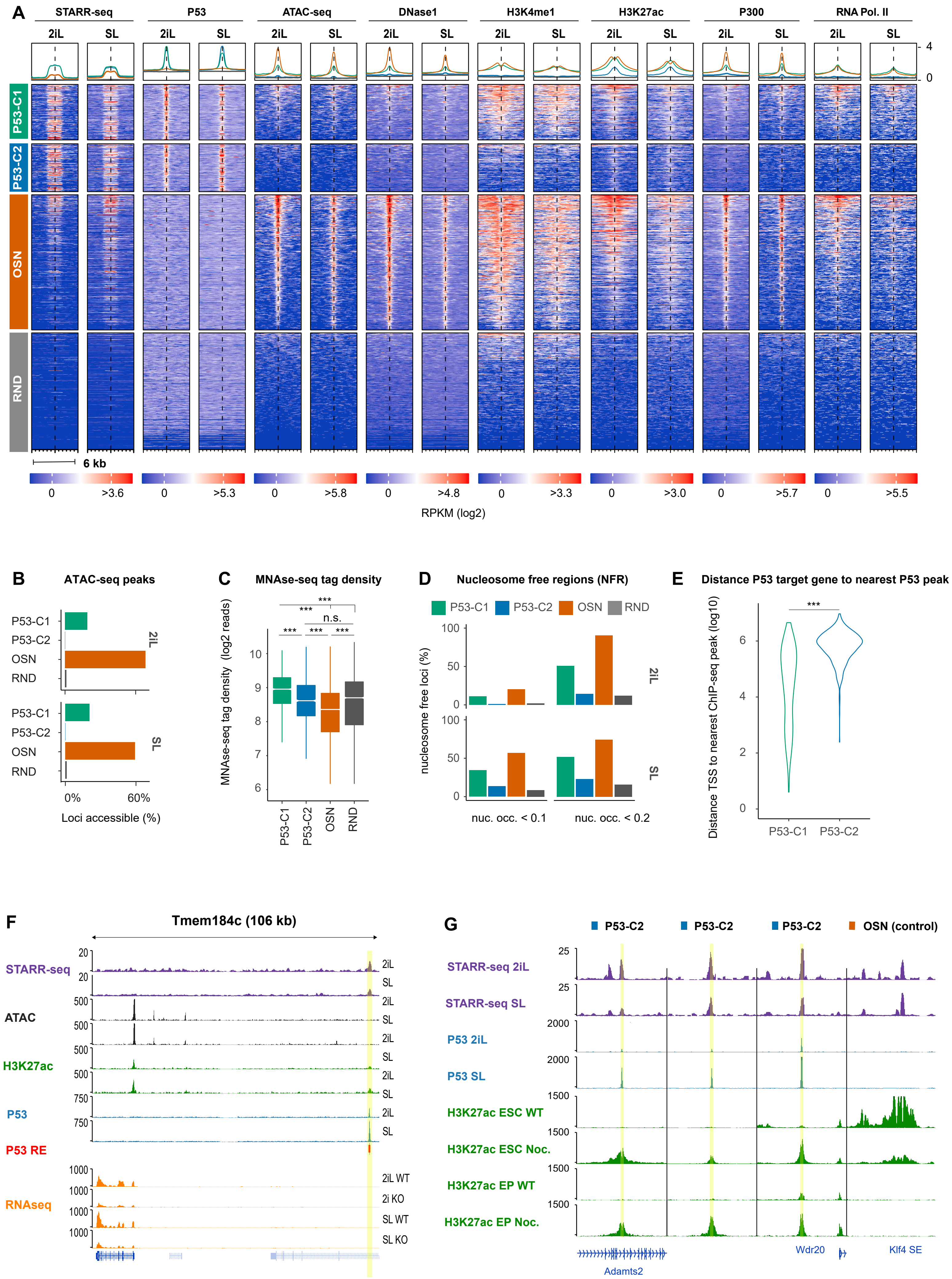
A
STARR-seq
P53
ATAC-seq
DNase1
H3K4me1
H3K27ac
P300
RNA Pol. II
2iL
SL
P53-C1
P53-C2
OSN
RND
6 kb
>3.6
>5.3
>5.8
>4.8
>3.3
>3.0
>5.7
>5.5
RPKM (log2)
B
ATAC-seq peaks
Loci accessible (%)
0%
60%
C
MNAse-seq tag density
MNAse-seq tag density (log2 reads)
n.s.
***
D
Nucleosome free regions (NFR)
nucleosome free loci (%)
nuc. occ. < 0.1
nuc. occ. < 0.2
E
Distance P53 target gene to nearest P53 peak
Distance TSS to nearest ChIP-seq peak (log10)
F
Tmem184c (106 kb)
STARR-seq
ATAC
H3K27ac
P53
P53 RE
RNAseq
2iL WT
2i KO
SL WT
SL KO
G
P53-C2
OSN (control)
STARR-seq 2iL
STARR-seq SL
P53 2iL
P53 SL
H3K27ac ESC WT
H3K27ac ESC Noc.
H3K27ac EP WT
H3K27ac EP Noc.
Adamts2
Wdr20
Klf4 SE

SUPPLEMENTARY FIGURE LEGENDS

*Some of the panels in these supplemental figures contain public data. These panels are annotated with **[PD]**. The accession numbers of public data and their corresponding panels are annotated in Additional file 2: Table S1.*

Figure S1: Quality assessment and chromatin-based classification of STARR-seq (lacking) loci.

A. Genome wide coverage and depth per STARR-seq input library.

B. STARR-seq enrichment at MACS2 peaks and randomly selected GC%-matched regions. A 3-fold or higher STARR-seq enrichment over input (dashed line) was observed in less than 3% of the randomly selected regions and used as a cutoff for significant enhancer signal.

C. Scatterplot of STARR-seq signal in biological replicate (rep.) 1 vs 2. PCC: Pearson's correlation coefficient.

D. Distribution of the GC% of DNA at STARR-seq peaks compared to ChIP-seq input libraries. GC% bias was computed from the mapped BAM files in 100bp windows across the mouse genome (see methods).

E. Number of STARR-seq peaks detected after merging the biological replicates and subsampling. The expected number of enhancers converges when ~60% of the reads in the merged libraries are sampled. Error bars denote the standard deviation of n=3 random subsamples.

F. **Left:** EpiCSeg chromatin segmentation of the mm9 genome into 5 clusters. Group 1-3 have active promoter/enhancer marks that are lacking at group 4-5. STARR-seq peaks overlapping a group 1-3 region are classified as C1 and largely overlap with the "enhancer" segments. STARR-seq regions that only overlap group 4-5 regions are classified as C2 and largely comprise "empty" segments enriched only for H3K9me3. For better visibility, columns were Z-scored and negative values were set to 0. **Right**: number of STARR-seq peaks assigned to each chromatin segment in 2iL or SL **[PD]**

G. Scatterplot of the ATAC-seq and STARR-seq enrichment at the C1-loci. ATAC-seq signal was computed at the STARR-seq summit flanked by 250bp on either side. PCC: Pearson's correlation coefficient. Ten red dots mark the values of the examples shown figure S1I.

H. Scatterplot of the H3K27ac and STARR-seq enrichment at C1-loci. H3K27ac signal was computed at the STARR-seq summit flanked by 2kb on both sides. PCC: Pearson's correlation coefficient. Ten red dots mark the values of the examples shown figure S1I.

I. Genome browser screenshots for C1 STARR-seq peaks with different levels of ATAC-seq, H3K27ac and STARR-seq enrichment. APK peak locations are indicated in the bottom (example 1-4). C1 STARR-seq peaks without a significant APK peak still show low

enrichment for H3K4me1, P300 or H3K27ac, but often lack a significant chromatin accessibility peak (example 5-10). Windows of 15kb are shown, centered on the STARR-seq peaks. See Additional file 3: Table S2 for genomic coordinates of these example loci.

J. Boxplots of the number of input DNA fragments per class shows that the STARR-seq negative C3-loci have an equal or higher input count compared to the STARR-seq positive loci.

K. Boxplot of the GC-content of C1-C3 loci (summit ± 250 bp). *** $p < 2e-16$, Wilcoxon rank-sum test.

L. Percentage of C1-C3 loci that overlap a CpG island annotated by the UCSC Table Browser (mm9). The overlap with the C2-class is below 1%.

M. Percentage of C1-C3 loci that overlap a TSS annotated by the Phantom5 consortium.

Figure S2: TCF3 and ZIC3 are associated with STARR-seq peaks induced in 2iL- and SL-ESCs respectively.

A. The strength of a STARR-seq peak correlates significantly with the coverage depth of the input library. This property is taken into consideration when calling differential STARR-seq peaks (see methods). Marginal densities are shown in green. PCC: Pearson's correlation coefficient.

B. STARR-seq and ATAC-/ChIP-seq signal at 2iL- (n=1,442) and SL-induced (n=3,688) STARR-seq peaks of class C1. P-values: Wilcoxon's rank-sum test (two-sided) **[PD]**

C. Examples of STARR-seq peaks with elevated signal in 2iL-ESCs. These loci are more accessible and have higher H3K27ac in 2iL-ESCs. TCF3 occupies these loci in SL-ESCs. The location of the luciferase primers (orange; see figure S2D and table S3) are shown in the bottom. **[PD]**

D. Luciferase signal (Firefly/Renilla) scaled to F/R of a control region in WT and *Tcf3*$^{-/-}$ ESCs for the five loci shown in figure S2C. Notice that the x-axis for SL is 10-fold lower compared to that of 2iL. See Table S3 for the genomic location and sequences of the primers that were used.

E. RNA-seq expression of *Tcf3* (orange), *Zic2* (blue) and *Zic3* (green) during the transition from 2iL- to SL-ESCs and at SL cells that were converted to EpiLCs for 72h. Error bars denote the standard deviation of biological duplicates per time point.

F. Union of ZIC3 binding sites in 2iL- and SL-ESCs (n=11,796). The number of peaks with significant STARR-seq enrichment ($FC \geq 3$ and $p < 0.05$; i.e. STARR-seq positive peaks) is annotated for promoter proximal and distal binding sites.

G. Scatterplot of the STARR-seq signal at STARR-seq positive ZIC3 binding sites. The signal is highly skewed towards SL-ESCs and all differential STARR-seq peaks ($|FC| > 2.5$, $p < 0.05$; DESeq2) are found in that condition.

H. Number of differentially expressed genes (DEGs, $|FC| \geq 2.5$ and $p < 0.05$) in *Zic3*$^{-/-}$ vs WT ESCs.

I. Browser screenshots of loci around genes annotated in figure 2L. The naïve pluripotency genes *Nanog* and *Prdm14* are expressed in 2iL- and SL-ESCs and have ZIC3-bound STARR-seq peaks (highlighted in yellow) in both conditions. *Lefty1*, *Emb* and *Gbx2* are cluster markers of SL-ESCs that lose expression upon genetic ablation of *Zic3*. These genes are associated with SL-specific STARR-seq peaks occupied by ZIC3. **[PD]**

Figure S3: C2-loci are active STARR-seq regions due to the absence of DNA methylation and repressive histone modifications.

A. Boxplots of the average CpG methylation at C1-, C2-, C3-loci (summit ±250bp) and 50,000 CG%-matched random regions (RND) in 2iL- and SL-ESCs. *** $p < 2e-16$, Wilcoxon rank sum test. **[PD]**

B. TF motifs whose presence (“yes” or “no”) is predictive of increased or decreased CpG-methylation at all STARR-seq peaks (n=25,616). CpG-methylation was measured in SL-cultured ESCs. STARR-seq peaks with a NRF1 or canonical P53 motif have on average 15-17% higher CpG-methylation in SL-ESCs compared to STARR-seq peaks lacking this P53 motif. Similarly, STARR-seq peaks with a ZIC3 motif have on average 14% less CpG-methylation in SL-ESCs than those lacking the ZIC3 motif. Average CpG-methylation was computed at the STARR-seq peak summit ± 250bp. CpG-methylation changes were computed by single motif linear regression. P-values: t-test, corrected for testing n=392 motifs (Benjamini-Hochberg). **[PD]**

C. CpG-methylation at C1 and C2 STARR-seq peaks in WT- and DNMT TKO ESCs cultured in SL. **[PD]**

D. H3K9me3 at C1-C3 loci and 10,000 randomly selected regions. Read coverage was computed in tiles of 50bp in a region that flanks the STARR-seq summit (C1, C2) or ATAC-seq peak (C3) by 3kb on both sides. The average intensity (log2 RPKM) is shown in the top of the figure.

E. H3K27me3 at C1-C3 loci and 10,000 randomly selected regions. Read coverage was computed in tiles of 50bp in a region that flanks the STARR-seq summit (C1, C2) or ATAC-seq peak (C3) by 3kb on both sides. The average intensity (log2 RPKM) is shown in the top of the figure.

F. Percentage of C1, C2, C3 or randomly sampled regions that intersect a H3K27me3 (top) or H3K9me3 (bottom) peak in 2iL or SL. For C1-, C2- and C3-loci we used the peak summit flanked by 250bp on both sides. 10,000 GC%- and size matched regions were sampled from the mouse genome.

Figure S4: P53-bound STARR-seq loci are characterized by low chromatin accessibility and are categorized into active (class C1) and inactive enhancers (class C2) based on the presence/absence of active histone modifications.

A. STARR-seq, P53 occupancy and a number of enhancer marks at P53-C1, P53-C2, OSN-bound and randomly selected loci. Notice that P53-C2 loci lack all enhancer marks except STARR-seq, whereas P53-C1 regions have H3K4me1, H3K27ac and Pol II, but are also minimally accessible. The heatmap was sorted by descending ATAC-seq signal (2iL). The color gradient depicts the RPKM (log2) of reads in tiles of 50bp. **[PD]**

B. Percentage of P53-C1, P53-C2, OSN-bound or randomly selected loci that intersect an ATAC-seq peaks (IDR < 0.03). **[PD]**

C. MNAse-seq nucleosome summit values (computed with Danpos2) for the nucleosome closest to a P53-C1, P53-C2, OSN- or randomly selected peak. For P53-C1 and P53-C2 the nucleosome with highest MNAse-seq signal within 74bp from the canonical P53 motif were considered. For OSN- and randomly selected regions, the highest signal within 74bp from the peak center was used. *** p < 2e-16, n.s.: not significant; Wilcoxon rank-sum test. **[PD]**

D. Percentage of P53-C1, P53-C2, OSN- and randomly selected loci for which the peak summit overlaps a nucleosome free region (NFR) in 2iL- or SL. NFRs were computed using NucleoATAC with the default settings (occ=0.1) and a relaxed (occ=0.2) nucleosome occupancy (nuc. occ.) cutoff (see methods).

E. Distance distribution of P53 target genes (FC < -2.5 and p < 0.05 in *Trp53*$^{-/-}$ vs WT; DESeq2) to the nearest P53-C1 or P53-C2 ChIP-seq peak. *** p < 2e-16, Wilcoxon rank-sum test.

F. *Tmem184c* loses expression in *Trp53*$^{-/-}$ compared to WT ESCs. The distal P53 binding site (yellow highlight) has minimal ATAC-seq accessibility compared to the *Tmem184* promoter region, but is (lowly) enriched for H3K27ac **[PD]**

G. P53-C2 STARR-seq peaks that gain H3K27ac after treatment with Nocodazole (Noc.) in ESCs and erythrocyte progenitors (EP). The OSN-bound superenhancer near *Klf4* (OSN control) is an active C1-enhancer that is not bound by P53. This enhancer loses, rather than gains H3K27ac after treatment with Nocodazole.
