# Supplementary material for: STARR-seq identifies active, chromatin-masked, and dormant enhancers in pluripotent mouse embryonic stem cells
Source: Genome Biol. 2020 Sep 10;21:243. doi: 10.1186/s13059-020-02156-3 (PMC7488044; doi:10.1186/s13059-020-02156-3)
Supplement: Supplementary file 8 — Additional file 8. Review history. [file 13059_2020_2156_MOESM8_ESM.docx]

**Review history**

**First round of review**

**Reviewer 1**

In this manuscript, Peng and co-workers make use of STARR-seq to identify potential enhancer activity in ESCs. To do this they compare differences in control mechanisms associated with differential gene expression in a line of ESCs cultured in two well-studied distinct regimes: one (SL) in which they exhibit heterogeneous (metastable) properties, the other (2iL) where they are more homogeneous and closely representative of the in vivo periimplantation epiblast. They first establish 3 major peaks and probe these to investigate differences in chromatin accessibility, transcription factor binding and histone modifications (APK elements). There was small mutual overlap and a large number of STARR-seq peaks not covered by the APK elements between the two culture conditions. 22 ChIP-seq datasets were collected from cells cultured in the two conditions. Most of the STARR-seq peaks >18,000 overlap genomic loci thought to be active enhancers (C1); ~7,000 have no active chromatin signature, but do show enhancer activity (C2) and ~2,000 show no enhancer activity (C3). Enhancer activity is measured by luciferase reporter assays. The 3 Cs are separable by TF motif, with C1 having naïve pluripotency genes, C2 enriched for P53, NRF1 and forkhead factors, whereas C3 has a more primed pluripotency motif, including ZIC3, and lacks naïve factors. No activity was seen in ZIC3 enhancers specific to 2iL, but plenty were specific to SL, suggesting ZIC3 is not important for maintaining naïve pluripotency in 2iL. Accordingly, ZIC3 KO in ESCs had no effect on 2iL cultures, but flattened the SL colonies and caused differentiation. Furthermore, enhancer activity was lost in SL, but not 2iL in Zic3-/- cells.
ScRNAseq was performed on Zic3-/- versus WT ESCs to look in detail at the proportion of cells becoming endoderm in SL. Marker expression suggested that the differentiated cells in the SL condition are endoderm. Methylation at enhancers is higher in C2 than C1, likely because of the detected elevated P53 and NRF1 in C2 loci. DNMT genes are collectively responsible for CpG methylation, but in DNMT TKO ESCs NRF1 occupancy is not much reduced, so there must be an alternative mechanism. H3K9me3 and H3K27me3 were investigate. C2 loci have significantly more H3K9me3 compared to C1 and C3, but negligible H3K27me3, confirming C2 loci inaccessibility compared with C1 or C3. Enhancer activity was indicated at some loci in C2, revealed specifically by STARR-seq. P53 presence is elevated in absence of H3K9me3 in C2 loci (no active chromatin). Also, P53 was seen in C1 where there are chromatin marks. P53 ChIP-seq was used to show that C1 loci are enriched for H3K4me1, H3K27ac and RNA Pol II, but not in C2 STARR peaks, although both groups showed low chromatin accessibility. Instead, nucleosome occupancy was seen more at P53 peaks than at OSN. To explore the role of P53 in gene expression, Trp53-/- ESCs were used. This revealed 888 differentially expressed genes in KO compared to WT, which turned out to be those closer to the TSS of P53 in C1 loci. H3K27ac was found to be induced during cellular stress and reprogramming. In conclusion, C1 loci are enriched for active enhancer marks that interact with target promoters via chromatin looping, whereas C2 loci lack enhancer marks that normally cause binding to promoters, but it can be forced under certain conditions.
This is a very well conceived and executed study of interest to those in the field of stem cell and developmental biology that reveals some interesting phenomena associated with the different phenotypes of ESCs between culture in SL or 2iL. It also defines a role for ZIC3 in protection of the state of naïve pluripotency in sub-optimal (SL) conditions.

Points for correction/clarification
1.     The sentences at the start of the introduction currently imply that propagation indefinitely in the pluripotent state is a feature of embryos (as well as ESCs), but this is not the case. They therefore need to be rewritten to emphasise that it is pluripotency, not self-renewal, that is a feature of early mammalian development.
2.     The sentence from line 37 to 40, on P8 seems to over-interpret the data in Fig. 2G. KLF2 and PRDM1 do appear significantly downregulated, but the other naïve factors are only marginally reduced. How is the significance measured?
3.     H3K7ac should be H3K27ac on line 60 of P11.
4.     Fig. 4C is difficult to understand.
5.     Refs 38 and 39 are duplicates, likewise 51 and 75.

**Reviewer 2**

This study aims to functionally identify and characterize transcriptional enhancers in "ground state" (2iL) and "metastable" (SL) murine ESCs on a genome-wide scale. To measure enhancer activity, the authors use STARR-seq, which assesses enhancers independently of their endogenous chromatin configuration, including silent or repressed enhancers. This identifies a total of ~25,000 enhancers (2 biological replicates; PCC>0.8) in mESCs under 2iL and SL culture conditions. Intersecting these candidates with publicly available datasets for DNA accessibility, histone modifications and TF and COF occupancy, enabled the authors to define two major classes of enhancers that are separated by active (C1) and inactive (C2) chromatin plus enhancers that were not active in STARR-seq (C3; APK only). In contrast to the active chromatin at C1 enhancers, the inactive chromatin at C2 enhancers appears more heterogenous, allowing to further subdivide into dormant and epigenetically repressed or chromatin masked enhancers.

C1 enhancers overlap "active chromatin" in a culture condition specific manner and are enriched for pluripotency factor motifs, further suggesting that they represent endogenously active enhancers. Differential analysis allowed the authors to identify TF motifs that are enriched in culture condition specific enhancers, which they exemplify with the pluripotency factor ZIC3 (SL-ESC specific). They profile ZIC3 binding by ChIP-seq and show that ZIC3 occupied regions exhibit enhancer activity specifically in SL-ESCs. Further, CRISPR mediated deletion of ZIC3 lead to the loss of enhancer activity in SL- but not 2iL ESCs, as shown by luciferase assays at selected loci in Zic3 KO ESCs. In addition, Zic3-/- ESCs show differentially expressed genes in SL but not in 2iL and a small fraction of Zic3-/- ESCs differentiates towards an endodermal cell fate when cultured in SL, as revealed by scRNA-seq. Through this comprehensive and well thought series of different experiments the authors show the cell-type specific importance of the pluripotency factor ZIC3 for enhancer activity and stability of the pluripotency network in SL-ESCs.

C2 enhancers overlap inactive or repressed chromatin, including regions that are repressed by DNA methylation or H3K9me3 (including regions enriched for IAP retrotransposons). This class also contains P53 bound regions, lacking active chromatin marks in ESCs, but gaining active marks under certain conditions (UV-irradiation, Nocodazole) and reprogramming of MEFS to iPSC, therefore termed dormant enhancers by the authors.

Using luciferase assays, enhancer activity could be validated for C1 and C2 enhancers but not C3, suggesting that C3 (APK) loci don't have enhancer activity.  

To confirm their classification of regulatory elements associated with different functions and TF dependencies (inferred from specific motif enrichment) of STARR-seq enhancers the authors decided to genetically knock out the respective factors (CRISPR KO cell lines) and investigating the consequences/impact on enhancer activity (luciferase assays) gene expression (RNA-seq) TF occupancy (ChIP-seq), as well as under unperturbed conditions again using publicly available data sets. This thoroughly planned experimental setup allowed the authors to identify enhancers that are active in mESCs as well as specific to either growth condition.

They also identify TFs that are involved in the regulation of these enhancers, namely confirm that TCF3 represses and identify ZIC3 that activates enhancers specifically in SL. They also identify enhancers that are endogenously repressed by DNA methylation, which prevents TF binding. ERVs that have enhancer activity are endogenously repressed by heterochromatin (H3K9me3). Finally, they define dormant enhancers, that are characterized by inaccessible chromatin but P53 occupancy and their ability to become active under certain conditions. Also, here the authors show nicely through a series of different experiments (incl TP53 KO ESCs) the importance of P53 for enhancer function of this class.

Overall, the paper contains many datasets representing a lot of work, is well structured, and the specific findings and the key message (different classes of enhancers, although broad and heterogeneous) is carefully experimentally validated. The work sheds more light on active and repressed regulatory elements in two different ES cell states that should be useful for the community. However, there are some critical points that the authors need to address by improving and clarifying text and figures (additional experiments are not necessary in our opinion).

Major concerns

1.     The authors use an old STARR-seq setup that has been updated recently (Muerdter et al. 2018) because of transcription initiation from the bacterial ORI and the mounting of an interferon response upon plasmid transfection in the majority of mammalian cell lines (leading to false positive and false negative enhancer activities). While the data should be of good enough quality even with the old setup (and it is unreasonable to repeat the experiments), the authors should discuss these issues and whether and how they might confound their data. From the data presented here it is difficult to estimate the real quality of the data, especially for enhancers in the lower range of enrichments. Maybe the authors could show more example screenshots of lower ranked enhancers to visualize the quality of peaks at different ranks.
2.     Please clarify if the peak calling is done on both sets of enhancers (2iL vs SL) separately or on the merged data from both conditions. Peak calling should ideally be done on each condition separately before merging the peaks for further analysis.
3.     The chromatin segmentation approach classifies a substantial number of enhancers as overlapping "active chromatin". From the presented data it is unclear how many C1 enhancers are associated with which chromatin feature. Also, the chromatin features of the luciferase validated C1 enhancers is unclear. What is the minimal requirement for "active chromatin"? The authors should provide numbers on that (e.g. included into Fig S1) and show more example screenshots for e.g. enhancers that are called active but are only bound by one TF and lack ATAC-seq signals or similar.
4.     The impact of culture conditions on the C2 enhancers is not clear. Also, throughout the paper we find it hard to see which mESC was used and why (does not apply to Figure 1)
5.     Introduction: The description of MPRAs (page 2-60 - 3-1) does not include the STARR-seq setup used. We therefore suggest to either write "by combining genomic fragments and a minimal promoter" or "in front of a minimal promoter or in the 3'-UTR of the reporter construct" or similar. Please also adjust the sentence and references: "Genome-wide STARR-seq has first been applied to Drosophila39-41 and mammalian cells42,43, including human ESCs enriched for active histone modifications44". Ref 42 describes CapSTARR-seq which uses a comprehensive library of pre-selected regions similar to ref 44. We therefore suggest citing Ref 43 and Muerdter et al. 2018 for genome-wide STARR-seq in mammalian cells and Refs 42 and 44 to acknowledge targeted approaches as in "genome-wide sets of preselected candidate regions" or similar.
6.     The methods section needs to be more detailed and checked for correctness. The methods should contain all critical information and parameter that would allow reproduction of the experiments. The current status of the methods section is far too superficial and needs significant improvement.
7.     The same applies to the figure legends. They appear very immature and lack important information (see also below).  To a lesser extent it also applies to the labeling of the figures itself.


Specific comments
(comments to the main text (per FIGURE X section) are shown first followed by comments per figures and respective legends)


MAIN TEXT:

FIGURE 1:
Were the STARR-seq peaks called on merged replicates? Please clarify (see also 2.).
Fig 1B: please add the genomic distribution for 2iL and SL separately (could be in Fig S1)
p4-16: this sounds like APK overlapping STARR-seq peaks were not included. Is this true?

FIGURE 2:
p5-4: "GACE" should be "CAGE"
p5-27: are all peaks from any condition used to compare only STARR-seq signals? Otherwise the analysis might be circular - please clarify!
p6-15: Fig. S2I-J should be a main figure - key validation! (bottom chart is relevant, results shown in top panel are unclear (not needed for main figure) - if displayed adjust scales for both panels)
Is TCF3 expressed in 2iL-ESCs?
p6-1&2: why are the ZIC3 ChIP-seq data sets for both conditions merged? Please clarify
p6-15: is there an explanation for increased luciferase activity in 2iL in the absence of ZIC3 (Fig. S2I-J)?
p6-37: 2iL RNA-seq data are not shown but referenced for Fig 2F, or is it wrong figure referencing? (Fig 2G?).
p7-12: please state the condition for the transcriptional profile.

Is the small fraction of SL-ESCs that adapts endodermal fate enough to explain "dramatic" upregulation in bulk RNA-seq? Please comment.

FIGURE 3:
p7-38: typo? C1 ◊ C2
Fig S3C: why is the WT methylation in C1 much higher than in C2?
p8-p10: inaccessible and H3K9me3 marked peaks and their enrichment in TPs has already been reported by Muerdter et al. 2018. Please add citation.

FIGURE 4:
p9-7: P53 binding is evaluated for the union of ChIP-seq peaks in 2iL and SL ESC peaks - please explain why the union is used
p9-60: typo H3K7ac ◊ H3K27ac & repgrogramming ◊ reprogramming
p10-1: wrong Fig referencing: Fig 4F ◊ 4H?
p10-7: there is no data that support the claim: "interact with target promoters through chromatin looping". Please clarify!

DISCUSSION:
p10-22: what overlap was anticipated and why?
p10-39: Fig 5 provides a summary of the three types of elements but without a legend is unclear. We recommend to include a legend, both in the figure itself (to e.g. explain the different symbols used) and an additional figure legend.
p10-47: regarding the reproducibility between replicates: please state that only technical replicates rather than biological replicates are shown
p11-49: chromatin looping - where are the data to this statement? Please clarify!!!

FIGURES & LEGENDS (L=legend; F=figure)

FIGURE 1:
L1C: were APK peaks defined as present in both 2iL & SL? Please clarify.
F1D: green arrow for C1, SL - what does this indicate?
L1F: "Next, the 39…" does not fit here. Should this not be L1G?
L1F: please define the error bars?
F1G: there are no error bars shown - but stated in legend?
F1G: please state more clearly that only technical replicates are provided for the luciferase tests
F1G: why was only one C2 enhancer validated per condition?
F1H: please explain why the motif enrichment has been done on the condition-pooled set of enhancers

FIGURE 2:
L2C: "Tcfcp2l1 gene that is higher expressed in 2iL-ESCs" - please add a reference
F2D: SL Zic3-/- ◊ are these cells shown at a different scale or are they indeed much smaller? Please clarify and also indicate the flattened-out ones.
F2G: why are there 2 columns for Zic3-/-? Are these replicates? Please clarify.
Please also clarify what is actually shown, raw reads or something else?
L2I: please expand the legend. Mention groups 1-4 that are indicated as colored boxes at the top.

FIGURE 3:
F3A: please add numbers.
F3B: what does DE mean?
F3D: what does C2-CO mean?
F3F: C2-CO ◊ please provide a number.

FIGURE 4:
L4A: is p=0.02 the p-value for SL? Please spell out SEM.
F4F: axis labels are unclear. Please clarify.
F4H y-axis: typo? C1◊C2

FIGURE 5:
We think that a more detailed figure legend is needed, as the figure is not as clear as probably intended (see also above). For example, what do the colors mean: Do the black or rather the transparent circles display DNA methylation? Does the distance between the nucleosomes mean something?

FIGURE S1:
FS1A: please explain and also specify "BR" (presumably biol replicate).
LS1C: please specify the color code.
FS1F: how many STARR-seq enhancers fall into each category?
Where are the data sets from?

FIGURE S2:
FS2A: is strength not STARR-seq over input? How does this compare to Fig S1C?
FS2B: while the trend is clear and supports the authors' statement, please add if the specific changes are significant
FS2D: x-axis labeling - please mention in the legend that the scale is different for 2iL and SL.
FS2F: please match the legend to the presented data (clone 1 & 2?)
FS2G: please explain why the union of peaks between conditions is used, especially given the condition-specific function of Zic3
FS2I: ZIC3 binding in both conditions?
FS2J: mention different scale of x-axis?


FIGURE S3:
FS3B: the legend needs to be more precise (Average ◊ average). Indicate SL in the figure.
FS3C: please indicate SL in the figure.
Why is C1 WT CpG methylation that high?

FIGURE S4:
FS4C: typo? x-axis P53-C2C2 ◊ P53-C2
FS4G: are this C1 enhancers?

REFERENCES:
#38 & #39 is the same reference

**Authors’ response to reviewers**

**Reviewer #1**: The reviewer accurately summarized our findings and concluded that “*This is a very well conceived and executed study of interest to those in the field of stem cell and developmental biology that reveals some interesting phenomena associated with the different phenotypes of ESCs between culture in SL or 2iL. It also defines a role for ZIC3 in protection of the state of naïve pluripotency in sub-optimal (SL) conditions.*”

**Points for correction/clarification**
*1.      The sentences at the start of the introduction currently imply that propagation indefinitely in the pluripotent state is a feature of embryos (as well as ESCs), but this is not the case. They therefore need to be rewritten to emphasise that it is pluripotency, not self-renewal, that is a feature of early mammalian development.*

The reviewer is correct and we have changed the sentences to emphasize pluripotency as the key feature of embryonic stem cells rather than self-renewal.

*2.      The sentence from line 37 to 40, on P8 seems to over-interpret the data in Fig. 2G. KLF2 and PRDM1 do appear significantly downregulated, but the other naïve factors are only marginally reduced. How is the significance measured?*

We measured significance using DESeq2 with the parameter estimateDispersion= “blind”, since we used 1 WT replicate and 2 Zic3KO replicates. Typically, this overestimates the dispersion and should thus result in less significant p-values (see: <http://bioconductor.org/packages/devel/bioc/vignettes/DESeq2/inst/doc/DESeq2.html#blind-dispersion-estimation>)

In the heatmap (figure 2G) the downregulation of *Prdm14* and *Klf2* is indeed most apparent, but the genes mentioned at line 37-40 are also downregulated by 2.4-fold or more. We added the fold changes and p-values on line 37-40 that now reads:

*“Simultaneously, ground-state pluripotency markers like Nanog (FC=-2.4, p=7.2x10^-9^), Klf2 (FC=-4.85, p=5.1x10^-25^), Tcfcp2l1 (FC=-3.4, p=5x10^-18^), Esrrb (FC=-2.4, p=1.4 x10^-9^) and most prominently Prdm14 (FC=-8.9, p=7.2 x10^-14^) are significantly downregulated in Zic3^-/-^ SL-ESCs, although most still are highly expressed (****Fig. 2G****).”*

Downregulation of *Nanog* is further apparent in the single cell analysis (top row of figure 2I), where it is a highly ranked gene (rank 39) that discriminates *Zic3*^-/-^ ESCs cultured in SL (Fig. 2H bottom, cluster 3; green) from their WT counter parts (Fig. 2H bottom, cluster 2; red) with an average fold change of 1.65 (adjusted p-value=2.4 x10^-23^). In comparison, *Prdm14* is downregulated by only 1.3 fold (adj p=6.4 x10^-4^) as its transcripts are only detected in a small portion of the WT cells cultured in SL (Fig. 2I, 4^th^ row) The downregulation of *Nanog* fits with an earlier report “*The Pluripotency Regulator Zic3 Is a Direct Activator of the Nanog Promoter in ESCs*, Stem Cells, 2010”. Our browser screenshot in Figure 2J further shows a ZIC3-bound promoter proximal and distal enhancer for *Nanog* with significant STARR-seq signal.

*3.      H3K7ac should be H3K27ac on line 60 of P11.*

Thank you, we corrected this.

*4.      Fig. 4C is difficult to understand.*

Figure 4C is a riverplot that shows that only ~10% of the P53-bound C1- and C2 STARR-seq peaks switch from a C1 to a C2 state between the 2iL and SL conditions or vice versa. Because 90% of the STARR-seq peaks remain a C1 or C2 between 2iL and SL, the flow may not be very apparent. We tried to visually optimize this figure and explain the main conclusion in the figure header in our revised manuscript. We also clarified what this panel represents in the figure legend.

*5.      Refs 38 and 39 are duplicates, likewise 51 and 75.*

We removed the duplicates, that likely occurred because the articles were loaded multiple times into the citation manager. We found that ref 22 and 91 (Habibi et al.) were also duplicated and the reference to BWA MEM (#79) was not well rendered. These issues have now been fixed, but we apologize for these mistakes, we clearly should have checked the bibliography more carefully.

**Reviewer #2**: The reviewer accurately summarized our findings and concluded that “*Overall, the paper contains many datasets representing a lot of work, is well structured, and the specific findings and the key message (different classes of enhancers, although broad and heterogeneous) is carefully experimentally validated. The work sheds more light on active and repressed regulatory elements in two different ES cell states that should be useful for the community. However, there are some critical points that the authors need to address by improving and clarifying text and figures (additional experiments are not necessary in our opinion).”*

We thank the reviewers for their time and the overall positive feedback on our study. Although we took care to report comparisons and statistics in our text and figure legends, we realize that we have not been careful enough. We addressed the detailed comments raised by the reviewers below and believe these have contributed significantly to the overall improvement of our manuscript. A detailed point-by-point response is provided below.

**Major concerns**

1.      The authors use an old STARR-seq setup that has been updated recently (Muerdter et al. 2018) because of transcription initiation from the bacterial ORI and the mounting of an interferon response upon plasmid transfection in the majority of mammalian cell lines (leading to false positive and false negative enhancer activities). While the data should be of good enough quality even with the old setup (and it is unreasonable to repeat the experiments), the authors should discuss these issues and whether and how they might confound their data. From the data presented here it is difficult to estimate the real quality of the data, especially for enhancers in the lower range of enrichments. Maybe the authors could show more example screenshots of lower ranked enhancers to visualize the quality of peaks at different ranks.

We agree that it is important to discuss the limitation and possible inaccuracies in the ‘old’ STARR-seq plasmids we used, but the data was already generated and partially analyzed and therefore not repeated (in line with the comments of the reviewer). Briefly, transcription initiation from the ORI will likely result in an underestimation of the STARR-seq signal and may have resulted in some false negatives. Importantly, it has been reported that mESC lack a type I interferon response^75, 76^. Therefore, we expect that our data is not affected by a confounding interferon response.

We wrote the following in the discussion section:

*“Note that we used an older version of the STARR-seq plasmid in which the bacterial origin of replication (ORI) functions as a (competitive) core promoter^42^. Transcripts initiated at the ORI may not reach the end of the luciferase gene or may not be accurately spliced, which would have resulted in an underestimation of the enhancer strength. In most mammalian cells, a second confounding effect is that DNA transfection induces a type I interferon response that gives rise to “false positive” enhancer elements^42^. Importantly, mESCs do not display this response^74,75^.”*

References

**^42^** Muerdter, F. *et al.* Resolving systematic errors in widely used enhancer activity assays in human cells. *Nat. Methods* **15**, 141–149 (2018).

**^75^** Wang, R. et al. Mouse embryonic stem cells are deficient in type I interferon expression in response to viral infections and double-stranded RNA. J. Biol. Chem. 288, 15926–15936 (2013).

**^76^** Witteveldt, J., Knol, L. I. & Macias, S. Microrna-deficient mouse embryonic stem cells acquire a functional interferon response. Elife 8, e44171 (2019).

We have included additional panels (**Fig. S1G, S1H and S1I)** to show the quality of our data. We show that ATAC-seq (Fig S1G) or H3K27ac (Fig S1H) are not strongly correlated with STARR-seq enrichment at C1-enhancers. Examples across the STARR-seq enhancer strength spectrum are shown in figure S1I. See point also point #3.

2.      Please clarify if the peak calling is done on both sets of enhancers (2iL vs SL) separately or on the merged data from both conditions. Peak calling should ideally be done on each condition separately before merging the peaks for further analysis.

We noticed that the term “merged” may have led to confusion here and in some of the subsequent questions regarding the ChiP-seq. In all cases, we identified peaks for each condition separately and took the union of these peaks using “*bedtools merge*”. We have described this more clearly in the methods section.

3.      The chromatin segmentation approach classifies a substantial number of enhancers as overlapping "active chromatin". From the presented data it is unclear how many C1 enhancers are associated with which chromatin feature. Also, the chromatin features of the luciferase validated C1 enhancers is unclear. What is the minimal requirement for "active chromatin"? The authors should provide numbers on that (e.g. included into Fig S1) and show more example screenshots for e.g. enhancers that are called active but are only bound by one TF and lack ATAC-seq signals or similar.

We used the tool EpiCSeg with condition-specific histone modification-, chromatin accessibility- and TF ChIP-seq data from our lab and others to classify chromatin segments as “active” or “inactive” (Fig. S1F, see Table S2 for accession numbers). We have now supplemented this panel with bar graphs that depict how many STARR-seq peaks fall into each category in 2iL or SL. One cannot easily quantify “a minimal requirement” for active chromatin, since the segmentation is based on a complex mixture of the marks. As is visible in our revised figure S1F, most C1 STARR-seq peaks have “classical” enhancer marks with enrichment for H3K4me1, H3K27ac, P300, Med1 and the core pluripotency factors OCT4, SOX2 and NANOG. The majority of C2 enhancers are void of all marks, except H3K9me3.

We included three additional panels in figure S1 that show that the strength of C1 enhancer elements does not correlate well with ATAC-seq (Fig S1G) or H3K27ac (Fig S1H), including examples across the STARR-seq enrichment spectrum (Fig S1I). Although many of the C1-enhancers lack an ATAC-seq peak, but there is an appreciable difference, particularly in ATAC-seq signal, between C1 enhancers with and without APK peak (see Fig S1I). We have not mentioned that these are bound by only one TF as remarked by the reviewer.

*4.      The impact of culture conditions on the C2 enhancers is not clear. Also, throughout the paper we find it hard to see which mESC was used and why (does not apply to Figure 1)*

We detected C2-enhancers in 2iL and SL-ESCs and the culture conditions have minimal effect on the enhancer strength and classification (see Fig 4C and the chromatin segmentation in Fig S1H). We explained more extensively when and why we investigated the STARR-seq in a condition specific or condition-agnostic manner.

*5.      Introduction: The description of MPRAs (page 2-60 - 3-1) does not include the STARR-seq setup used. We therefore suggest to either write "by combining genomic fragments and a minimal promoter" or "in front of a minimal promoter or in the 3'-UTR of the reporter construct" or similar.*

We thank the reviewer and have included the suggested sentence.

*Please also adjust the sentence and references: "Genome-wide STARR-seq has first been applied to Drosophila39-41 and mammalian cells42,43, including human ESCs enriched for active histone modifications44". Ref 42 describes CapSTARR-seq which uses a comprehensive library of pre-selected regions similar to ref 44. We therefore suggest citing Ref 43 and Muerdter et al. 2018 for genome-wide STARR-seq in mammalian cells and Refs 42 and 44 to acknowledge targeted approaches as in "genome-wide sets of preselected candidate regions" or similar.*

We agree with the reviewer and have adjusted the text and citations accordingly.

*6.      The methods section needs to be more detailed and checked for correctness. The methods should contain all critical information and parameter that would allow reproduction of the experiments. The current status of the methods section is far too superficial and needs significant improvement.*

We agree with the reviewer and have expanded our method section considerably.

*7.      The same applies to the figure legends. They appear very immature and lack important information (see also below).  To a lesser extent it also applies to the labeling of the figures itself.*

We thank the reviewer for the detailed feedback on our figure legends and have addressed all concerns raised below. We further checked carefully that all our figures and legends show and explain what is shown in a reproducible manner.

**Specific comments**
(comments to the main text (per FIGURE X section) are shown first followed by comments per figures and respective legends)

 *MAIN TEXT:

FIGURE 1:
Were the STARR-seq peaks called on merged replicates? Please clarify (see also 2.).*
We realize that the term “merged” may have led to confusion here and in some of the later comments. To clarify: peaks were called on individual libraries (binomial p < 0.05), with an additional criterium of 3-fold enrichment per condition. We agree that this may have been un clear from the methods section and have clarified that accordingly in the revised manuscript. We replaced the term merged with “union” wherever appropriate and used the term “merged” (between quotes) with a clear indication of what is meant.

*Fig 1B: please add the genomic distribution for 2iL and SL separately (could be in Fig S1)*
We divided figure 1B into 2iL and SL and indicated the number of enhancers in each condition, their intersection, union and the number of enhancer elements that is unique to 2iL- or SL-ESCs.

*p4-16: this sounds like APK overlapping STARR-seq peaks were not included. Is this true?*
This sentence is indeed confusing. The APK-peaks were included in the analysis. We changed the sentence p4-16 in our revised manuscript to:

*“This analysis showed that 75% (19,220 of 25,616) of our STARR-seq peaks are not intersecting an APK-element (Fig. 1C, upper diagram). Therefore, we decided to thoroughly examine evidence of chromatin accessibility, TF occupancy or histone modifications at all our STARR-seq peaks”*

*FIGURE 2:
p5-4: "GACE" should be "CAGE"*
Thank you, we have corrected the typo in the revised manuscript.

*p5-27: are all peaks from any condition used to compare only STARR-seq signals? Otherwise the analysis might be circular - please clarify!*

To be honest, we don’t really understand the issue raised by the reviewer or why the reasoning would be circular. In this analysis, we took the union of the STARR-seq peaks detected in 2iL or SL, using “*bedtools merge*”. We clarified this procedure in the methods section under the header *“Merging” of 2iL- and SL STARR-seq peaks.* Differential analysis was performed for the enhancers in this “merged” peak set using a DEseq2 count model that considers the input DNA reads and SCP1 promoter reads for each library (2iL rep1, 2iL rep2, SL rep1, SL rep2). In brief, this model estimates the change in enhancer strength for each peak that was detected in 2iL or SL (or both) and uses the variance between replicates and conditions to estimate significance.

p6-15: Fig. S2I-J should be a main figure - key validation! (bottom chart is relevant, results shown in top panel are unclear (not needed for main figure) - if displayed adjust scales for both panels)

We agree and moved the original figures S2I and S2J to the main (now revised figure 2F and 2G), together with the western blot from original figure S2F (revised figure 2D). We rescaled the luciferase bars for 2iL to the same values as for SL. In turn, we moved the browser screenshots (original figure 2J) to the supplement (revised figure S2I).

*Is TCF3 expressed in 2iL-ESCs?*

Yes, TCF3 (gene name *Tcf7l1*) is highly expressed both in 2iL- and SL-ESCs. We added the expression profile of *Tcf7l1* (revised figure S2E) and modified the sentence about TCF3 to:

*“TCF3 (Tcf7l1) is the highest expressed TCF/LEF member in ESCs, with similar expression in 2iL- and SL-ESCs (Fig. S2E). De-repression of TCF/LEF activity is a well-known effect of CHIR99021 mediated GSK3-inhibition^52–54^ that works through altered WNT-signaling, rather than modified Tcf7l1 expression levels.”*

*p6-1&2: why are the ZIC3 ChIP-seq data sets for both conditions merged? Please clarify*

Possibly the confusion again arises from the ambiguous term “merged”. Briefly, we called peaks with MACS2 followed by IDR to find reproducible ZIC3 binding sites in 2iL or SL. Next, we took the union of the two peak sets. We counted the number of STARR-seq and STARR input reads within each of these peaks for all STARR-seq libraries (2x 2iL, 2x SL). Next, we computed the STARR-seq enrichment in 2iL and SL and identified enhancers with differential strength (FC > 2.5, DEseq2 p-value < 0.05) using the aforementioned differential analysis. Looking only at the ZIC3 binding sites detected in one condition (e.g.) in SL would bias the STARR-seq enrichment towards that condition (SL). Therefore, we considered the union of 2iL and SL.

*p6-15: is there an explanation for increased luciferase activity in 2iL in the absence of ZIC3 (Fig. S2I-J)?*

We also noticed the increased luciferase activity in *Zic3*^-/-^ ESCs cultured in 2iL, but did not find conclusive evidence for a repressive function of ZIC3 in 2iL in our data or in the literature. Moreover, our RNA-seq data showed a minimal transcriptional response after loss of ZIC3 in 2iL. In particular, only 3 genes are significantly upregulated (FC ≥ 2.5, p < 0.05) in 2iL-cultured *Zic3*^-/-^ ESCs, suggesting that ZIC3 is not a potent repressor in 2iL-ESCs. Therefore, we focused on the activating function of ZIC3 in SL.

*p6-37: 2iL RNA-seq data are not shown but referenced for Fig 2F, or is it wrong figure referencing? (Fig 2G?).*

This is not what we intended to say, but we understand that this sentence is confusing. We modified the text to:

*“Marker genes of the primitive endoderm (PrE), like Sox7, Sox17, Gata4, Cubn and Dab2^56^ are all upregulated by 8-fold or more in SL (p < 2e-15 for all), in line with results reported for Zic3 RNAi ECSs cultured in SL^57^ (Fig. 2I). Interestingly, none of these genes are differentially expressed in 2iL-ESCs (data not shown).”*

*p7-12: please state the condition for the transcriptional profile.*
The transcription profile is for SL-ESCs. We have clarified this in the revised manuscript.

*Is the small fraction of SL-ESCs that adapts endodermal fate enough to explain "dramatic" upregulation in bulk RNA-seq? Please comment.*

We can estimate this from the single cell data. In our scRNA-seq, we have 234 WT SL-ESCs (cluster 2, red), 263 Zic3^-/-^ SL-ESCs that remain pluripotent (cluster 3, green) and 46 SL cultured Zic3^-/-^ SL-ESCs that adapt to an endodermal fate (cluster 4, orange). Since bulk RNA-seq expression is obtained from a large number of pooled cells, we sum the counts from single cells.

We inspected the endodermal markers: *Sox7*, *Sox17*, *Gata4* and *Gata6*. For the 234 WT-ESC (SL), we detected 8, 1, 18 and 98 transcripts respectively, indicating that basically these genes are not expressed in ESCs. For the 46 differentiated cells, we detected 547, 1632, 1937 and 5426 transcripts. The 263 Zic3^-/-^ cells in cluster 3 (green) do not express Sox7 (2 reads) or Sox17 (43 reads), but *Gata4* (153 reads) and in particular *Gata6* (1013 reads) are already upregulated compared to WT ESCs.

After correcting for sequencing depth differences, we find an upregulation of endodermal markers in cluster 4 (SL, differentiated cells) that exceeds 15-fold compared to cluster 2 (SL-WT ESCs). Thus, the “dramatic” upregulation of endodermal markers observed in the bulk RNA-seq data are indeed mostly coming from the ~15% of the cells that differentiate.

**Remark with respect to figure 1L**

We noticed that in Figure 1L (cluster markers) not the log2 fold change of the genes, but the Z-score (normalized expression values) are shown. In principle, this does not change the interpretation of the figure or the cluster markers, but the label in the figure was incorrect.

*FIGURE 3:
p7-38: typo? C1 ◊ C2*
Thank you, we fixed the typo

*Fig S3C: why is the WT methylation in C1 much higher than in C2?*
Thank you, we mistakenly swapped the labels for the C1 and C2 class. These methylation level should indeed have been similar to those shown in figure S3A. We repaired this in our revised manuscript.

*p8-p10: inaccessible and H3K9me3 marked peaks and their enrichment in TPs has already been reported by Muerdter et al. 2018. Please add citation.*

We apologize, we have included the following citation in our revised manuscript:

*“STARR-seq enrichment at H3K9me3 repressed ERVs has previously been reported in human HeLa-S3 cells (Muerdter et al. 2018). To examine whether our C2-loci are also enriched at ERVs, we intersected them with a list of established retrotransposons”*

*FIGURE 4:
p9-7: P53 binding is evaluated for the union of ChIP-seq peaks in 2iL and SL ESC peaks - please explain why the union is used*

We examined the role of P53-bound enhancers in 2iL and SL and found that the loci we tested are active in both conditions and lose this activity when P53 is lost (Fig. 4A). We detected that the number of P53-bound enhancers with significant STARR-seq activity is roughly similar in 2iL and SL (Fig 4B) and that only a small number switched from an active state (C1) in 2iL to an inactive state (C2) in SL or vice versa (Fig 4C). We focused follow-up experiments on what we regarded as the more striking observation that P53 occupies active and inactive enhancers in both pluripotent states.

We added the rationale to consider the union in our revised manuscript with the sentence

*“Given that we observed a similar loss of enhancer signal after genetic depletion of P53 in 2iL- and SL-ESCs (****Fig. 4A****), a similar fraction of P53-associated enhancers (****Fig. 4B****) and a largely unmodified epigenetic landscape (****Fig 4C****), we performed further analysis on the union of P53 binding sites detected in 2iL- and SL-ESCs.”*

*p9-60: typo H3K7ac ◊ H3K27ac & repgrogramming ◊ reprogramming*
Thank you, we fixed the typos

*p10-1: wrong Fig referencing: Fig 4F ◊ 4H?*
We intended to reference figure 4F, not 4H here. In the next sentence: *“This transient induction became even more apparent when we called H3K27ac peaks in two recent, independent iPSC reprogramming studies^74,75^ (****Fig. 4H-I****)”,* we examined intermediate reprogramming time point in more depth. However, the orange label in figure 4F should have read “reprogramming”, not “UV radiation”, which was duplicated. We apologize for this mistake and corrected it in the revised manuscript.

*p10-7: there is no data that support the claim: "interact with target promoters through chromatin looping". Please clarify!*
In an earlier draft of our manuscript we had compared chromatin looping using our previously published capture Hi-C data (Joshi et al. Cell Stem Cell, 2015). Since the capture probes were designed to target accessible DNA identified by DNAse1 hotspots, we realized (and confirmed) that this design was highly biased towards the C1-loci and thus could not be used to compare chromatin interactions between C1- and C2-loci. We thought we had removed these statements from the figures and results section, but clearly missed it here and in the discussion section. We have removed these statements in our revised manuscript.

*DISCUSSION:
p10-22: what overlap was anticipated and why?*

We revised the sentence and reported on the fact that 25% of the STARR-seq peaks overlap an APK-element and used EpiCSeg to better understand the chromatin state of the remaining 75%. We chose to stay closer to the facts and consequently have removed this subjective “*we anticipated...*” statement from the discussion.

*p10-39: Fig 5 provides a summary of the three types of elements but without a legend is unclear. We recommend to include a legend, both in the figure itself (to e.g. explain the different symbols used) and an additional figure legend*
Thank you. We have added an explanation of the symbols and an extended figure legend.

*p10-47: regarding the reproducibility between replicates: please state that only technical replicates rather than biological replicates are shown*
With this sentence we meant to say that the STARR-seq is reproducible between biological replicates. To make this distinction clearer, we modified this sentence into:
*“Our STARR-seq data was reproducible between biological replicates STARR-seq and validated by luciferase assays at selected loci”*
We believe that is not necessary to emphasize the technical replicates of the luciferase in this discussion, more so because the general correlation between STARR-seq and 43 validated loci was highly significant.

*p11-49: chromatin looping - where are the data to this statement? Please clarify!!!*
We apologize and have removed this statement. See our reply to *p10-7* regarding the Capture Hi-C data above.

*FIGURES & LEGENDS (L=legend; F=figure)

FIGURE 1:
L1C: were APK peaks defined as present in both 2iL & SL? Please clarify.*

We took APK-peaks and STARR-seq peaks that were present in 2iL ór SL (in both cases the union). The goal was to investigate whether STARR-seq peaks have an active chromatin signature in ESCs or not. We revised the legend to clarify the comparisons.

*F1D: green arrow for C1, SL - what does this indicate?*

The arrows indicated the origin of the examples in the heatmap, in a version of the heatmap that was sorted by the difference in STARR-seq signal between 2iL and SL. It is clear that they are more confusing then helpful and therefore we choose to remove them and label the classes of the examples in figure 1E. The additional examples shown in figure S1I should be helpful to the reader to appreciate the quality of our data.

*L1F: "Next, the 39…" does not fit here. Should this not be L1G?*

Thank you, this is indeed confusing. We clarified this in the legend and moved the scaling procedure to the methods section.

*L1F: please define the error bars?*
Thank you, these error bars were mistakenly added to L1G, which indeed does not have error bars.

*F1G: there are no error bars shown - but stated in legend?*
Thank you, this figure indeed has no error bars. The error bar part belonged to figure 1F.

*F1G: please state more clearly that only technical replicates are provided for the luciferase tests*
We clarified in the legend that the points represent the mean of two biological replicates for STARR-seq and technical triplicates for luciferase assays.

*F1G: why was only one C2 enhancer validated per condition?*
We initially validated enhancers with different levels of chromatin accessibility and H3K27ac. Later, we refined our analysis and separated enhancers into C1 and C2 classes. Although we examined several enhancers with (very) low accessibility and H3K27ac, these were later still classified as C1 enhancers. Reassuringly, we detected a strong increase of H3K27ac at P53-bound C2 enhancers in Nocodazole treated cells, which indicates that P53 can indeed induce these enhancers and they are not false positives.

*F1H: please explain why the motif enrichment has been done on the condition-pooled set of enhancers*

In this analysis we sought to find a comprehensive set of motifs that discriminate the C1, C2 and C3 classes. We now realize that this figure breaks the style with most of the previous panels in which we separated the 2iL and SL data. Therefore, we re-analyzed the motif enrichment in a condition-specific manner and replaced figure 1H. The results remain largely the same, but the 2iL-specifc TCF/LEF motifs and the SL-specific ZIC/ZFP281 motifs (Fig 2A and 2B) are now also visible in this figure.

*FIGURE 2:
L2C: "Tcfcp2l1 gene that is higher expressed in 2iL-ESCs" - please add a reference*
We added a reference to: Ye et al., Embryonic stem cell self-renewal pathways converge on the transcription factor Tfcp2l1, EMBO journal (2013).

*F2D: SL Zic3-/- ◊ are these cells shown at a different scale or are they indeed much smaller? Please clarify and also indicate the flattened-out ones.*

No, the same magnification is used as indicated by the bars. The vast majority of Zic3-/- cells have an evidently different morphology.

*F2G: why are there 2 columns for Zic3-/-? Are these replicates? Please clarify. Please also clarify what is actually shown, raw reads or something else?*

These are indeed biological replicates. The color scale denotes DESeq2 normalized counts (log2). We clarified this in the figure, which is figure 2J in the revised manuscript.

*L2I: please expand the legend. Mention groups 1-4 that are indicated as colored boxes at the top.*
We expanded the legend (now belonging to revised figure 2L) with the colored boxes and explained that the genomic loci for the genes color in blue or red are shown are in figure S2I.

*FIGURE 3:
F3A: please add numbers.*
We added the numbers in the figure.

*F3B: what does DE mean?*
Thank you, the abbreviation should have been D.O.. To avoid unclarity, we chose to write this out in the figure legend and removed the abbreviation from the figure caption. We also added the numbers

*F3D: what does C2-CO mean?*

These are the TF motifs that are co-occurring (main text). We clarified this in the figure legend. We further noticed that we used a minus sign on the y-axis (-log10 p-value) to denote statistical depletion (i.e. less often occurring than expected by chance). While it is convenient to code it this way, it is incorrect. We corrected this in this figure and in figure 3E and denoted “depletion” explicitly.

*F3F: C2-CO ◊ please provide a number.*
We added the numbers in the figure.

FIGURE 4:
*L4A: is p=0.02 the p-value for SL? Please spell out SEM.*

Yes, p=0.02 is the p-value for SL. We made this clearer and wrote SEM in full.

*F4F: axis labels are unclear. Please clarify.*

We clarified the labels and made the nomenclature (peaks, loci, regions) consistent.

*F4H y-axis: typo? C1◊C2*

Thank you, we corrected the typo and made the y-axis description in line with figure 4F.

*FIGURE 5:
We think that a more detailed figure legend is needed, as the figure is not as clear as probably intended (see also above). For example, what do the colors mean: Do the black or rather the transparent circles display DNA methylation? Does the distance between the nucleosomes mean something?*
We added an extended legend and explained the symbols in the figure. We made the distance between the nucleosomes equal, as these did not have special meaning.

*FIGURE S1:
FS1A: please explain and also specify "BR" (presumably biol replicate).*

We changed “BR” in the figure to “biol. replicate”.

*LS1C: please specify the color code.*

Thank you, the color gradient denotes the “level” of the contour plot and is associated with the density of the points. We added the color gradient to the figure in our revised manuscript.

*FS1F: how many STARR-seq enhancers fall into each category?*
We added histograms on the side of the figure that depict the number of peaks in each category in a condition-specific (2iL and SL) manner. Reassuringly, the vast majority of the C1 peaks that we called “enhancers” coincide with the chromatin segmentation class that we labeled “enhancers”. Similarly, the majority of STARR-seq peaks we termed “C2” only have enrichment for H3K9me3.

*Where are the data sets from?*
The origin of all the public data sets we used (indicated in the figure caption with **PD**) is specified in Table S2. We mentioned this at the top of the figure legends and supplemental figure legends with the sentence *“Some of the panels in these figures contain public data. These panels are annotated with* ***[PD]****. The accession numbers of public data and their corresponding panels are annotated in Table S2.”*

*FIGURE S2:
FS2A: is strength not STARR-seq over input? How does this compare to Fig S1C?*

Figure S1C shows the enrichment (after Bayesian shrinkage) between biological replicate 1 and 2. Figure S2A shows that at the 25,616 STARR-seq peaks, there is a significant linear relation between the amount of RNA transcribed from the SCP1 minimal promoter and the amount of input DNA sequenced from the transfected library. Since these are enhancers, this relation is expected. For differential analysis, one could compare the fraction RNA/input DNA at each site.

In our view, the better way to estimate differential enhancer strength is to use the count nature of the data explicitly using a DESeq2 model that accounts for input bias and estimates variance between conditions (2iL vs SL) and between biological replicates to estimate significant changes in enhancer “strength”. We implemented this differential analysis procedure as described in the method section.

*FS2B: while the trend is clear and supports the authors' statement, please add if the specific changes are significant*

We have added the p-values, which are all significant. For clarity, we also added the numbers in the figure itself.

*FS2D: x-axis labeling - please mention in the legend that the scale is different for 2iL and SL.*

We mentioned the scale difference in the legend.

*FS2F: please match the legend to the presented data (clone 1 & 2?)*
We matched the legend to the data.

*FS2G: please explain why the union of peaks between conditions is used, especially given the condition-specific function of Zic3.*

See our explanation at “*p6-1&2: why are the ZIC3 ChIP-seq data sets for both conditions merged? Please clarify”*

*FS2I: ZIC3 binding in both conditions?*

Correct, see explanation at “*p6-1&2: why are the ZIC3 ChIP-seq data sets for both conditions merged? Please clarify*”

*FS2J: mention different scale of x-axis?*

As advised at p6-15 above, we moved this figure to the main figures (figure 2G in the revised manuscript) and rescaled the x-axis for 2iL.

*FIGURE S3:
FS3B: the legend needs to be more precise (Average ◊ average). Indicate SL in the figure.*

We made the legend more precise and the x-axis more intuitive than a “regression coefficient”.

*FS3C: please indicate SL in the figure.*
SL was on the y-axis of the figure, but we now made this more explicit.

*Why is C1 WT CpG methylation that high?*
Thank you, this panel indeed does not match figure S3A. We check our code for both panels and confirmed that this panel shows an old figure with swapped C1 and C2 labels. We now included the correct figure in our revised manuscript. Similarly, we noticed that we had swapped the C1/C2 labeling for the nucleosome occupancy plot in figure 4D and corrected this in our revised manuscript.

*FIGURE S4:
FS4C: typo? x-axis P53-C2C2 ◊ P53-C2*

Thank you, we corrected the typo.

*FS4G: are this C1 enhancers?*

Thank you, these are indeed not C1, but C2 enhancers (except for the OSN-bound control region near *Klf4*). We missed this and corrected it in our revised manuscript.

*REFERENCES:
#38 & #39 is the same reference*

Thank you. we removed this and other duplicates we detected from the bibliography.

**Second round of review**

**Reviewer 2**

The authors have addressed all our concerns and present a manuscript that is well structured and reports many datasets and specific findings that are experimentally validated. The work sheds more light on active and repressed regulatory elements in two different ES cell states that should be useful for the community. We therefore recommend publication.
